# Enhancement of Ion Pairing of Sr(II) and Ba(II) Salts by a Tritopic Ion‐Pair Receptor in Solution

**DOI:** 10.1002/cphc.202000507

**Published:** 2020-08-13

**Authors:** Bence Kutus, Jun Zhu, Jian Luo, Qi‐Qiang Wang, Alexandru Lupan, Amr A. A. Attia, De‐Xian Wang, Johannes Hunger

**Affiliations:** ^1^ Department of Molecular Spectroscopy Max Planck Institute for Polymer Research 55128 Mainz Germany; ^2^ Beijing National Laboratory for Molecular Sciences CAS Key Laboratory of Molecular Recognition and Function Institute of Chemistry University of Chinese Academy of Sciences Chinese Academy of Sciences Beijing 100190 China; ^3^ Faculty of Chemistry and Chemical Engineering Babeş-Bolyai University 400028 Cluj-Napoca Romania

**Keywords:** cooperative effects, dielectric relaxation spectroscopy, host-guest systems, ion pairing, ion-pair receptor

## Abstract

Tritopic ion‐pair receptors can bind bivalent salts in solution; yet, these salts have a tendency to form ion‐pairs even in the absence of receptors. The extent to which such receptors can enhance ion pairing has however remained elusive. Here, we study ion pairing of M^2+^ (Ba^2+^, Sr^2+^) and X^−^ (I^−^, ClO_4_^−^) in acetonitrile with and without a dichlorooxacalix[2]arene[2]triazine‐related receptor containing a pentaethylene‐glycol moiety. We find marked ion association already in receptor‐free solutions. When present, most of the MX^+^ ion‐pairs are bound to the receptor and the overall degree of ion association is enhanced due to coordinative, hydrogen‐bonding, and anion‐π interactions. The receptor shows higher selectivity for iodides but also stabilizes perchlorates, despite the latter are often considered as weakly coordinating anions. Our results show that ion‐pair binding is strongly correlated to ion pairing in these solutions, thereby highlighting the importance of taking ion association in organic solvents into account.

## Graphical abstract

**Pairing in the spotlight**: Sr^2+^ and Ba^2+^ salts form ion pairs with iodide and perchlorate anions in acetonitrile. Adding a dichlorooxacalix[2]arene[2]triazine‐related ion‐pair receptor stabilizes these ion pairs and enhances the overall degree of ion pairing, as evidenced from their contribution to the dielectric loss spectra.

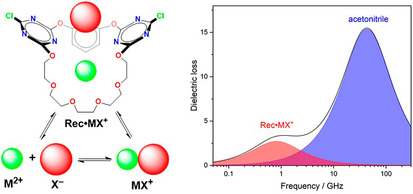

## 1 Introduction

Ion receptors have reached by now an elaborate design,^1^, ^2^, ^3^, ^4^, ^5^, ^6^ yet when coordinating a single ion, the corresponding counter‐ion affects both binding strength and selectivity. To also control the binding of the counter‐ion, ion‐pair (IP) receptors, which have cation and anion recognition moieties in the same molecular scaffold, have become the focal point of recent ion sensing studies.^7^, ^8^, ^9^, ^10^, ^11^ Since these receptors benefit from synergistic effects between the co‐bound ions, such as electrostatic and allosteric interactions, they exhibit enhanced binding affinities. Additionally, the modification of the recognition sites allows for fine‐tuning the selectivity, thus, a plethora of receptors has been designed for efficient binding of alkali metal (MX) and tetraalkylammonium salts (R_4_NX).^7^, ^8^, ^9^, ^10^, ^11^, ^12^, ^13^, ^14^, ^15^, ^16^, ^17^, ^18^, ^19^, ^20^, ^21^, ^22^, ^23^, ^24^, ^25^, ^26^, ^27^, ^28^, ^29^, ^30^, ^31^, ^32^, ^33^, ^34^, ^35^, ^36^, ^37^, ^38^, ^39^ As such, IP receptors have emerged as potential candidates for numerous applications, such as salt extraction,^12^, ^13^, ^14^, ^15^, ^16^, ^17^ transmembrane transport,^18^, ^19^, ^20^, ^21^, ^22^, ^23^ and catalysis.^24^, ^25^

The ability to tailor cationic and anionic binding sites also enables the design of multitopic receptors. In contrast to MX receptors, only a few structures that bind the cation and both anions of bivalent (MX_2_) salts, have been reported to date.^39^, ^40^, ^41^, ^42^ Such MX_2_ receptors could improve the extraction of alkaline metal earth cations, like Sr^2+^ and Ba^2+^,^43^, ^44^, ^45^, ^46^ from aqueous solutions or the selective extraction of the hazardous ^90^Sr from calcium‐containing radioactive wastes.^47^−^49^

The binding of MX_2_ salts to such receptors has been mostly derived from titration experiments. In such titrations typically the receptor is probed (NMR chemical shift or optical absorption/fluorescence), and thus interaction of individual ions with the receptor can be quantified given that ion binding results in salient variations of the receptor's chemical environment. In turn, it is challenging to detect weak interactions of the anion or cation of an IP with the receptor, and it is impossible to account for the formation of bare IPs that are not directly bound to the organic molecule. The latter is in particular relevant to bivalent MX_2_ salts, as they have a high tendency to form IPs in solution – i. e. cations and anions form long‐lived aggregates in solution – even in the absence of a guiding molecular scaffold.^50^ Thus, one fundamental question about the function of IP receptors has remained elusive: can these receptors efficiently bind pairs of ions and thereby enhance the overall degree of ion association? That is, does the formation of receptor‐IP complexes increase the overall concentration of associated ions (i.e. bare ion‐pairs and receptor‐bound ion‐pairs)? Quantifying this receptor‐induced enhancement of ion association can thus provide essential information about the function of such receptors in solution.

**Scheme 1:**
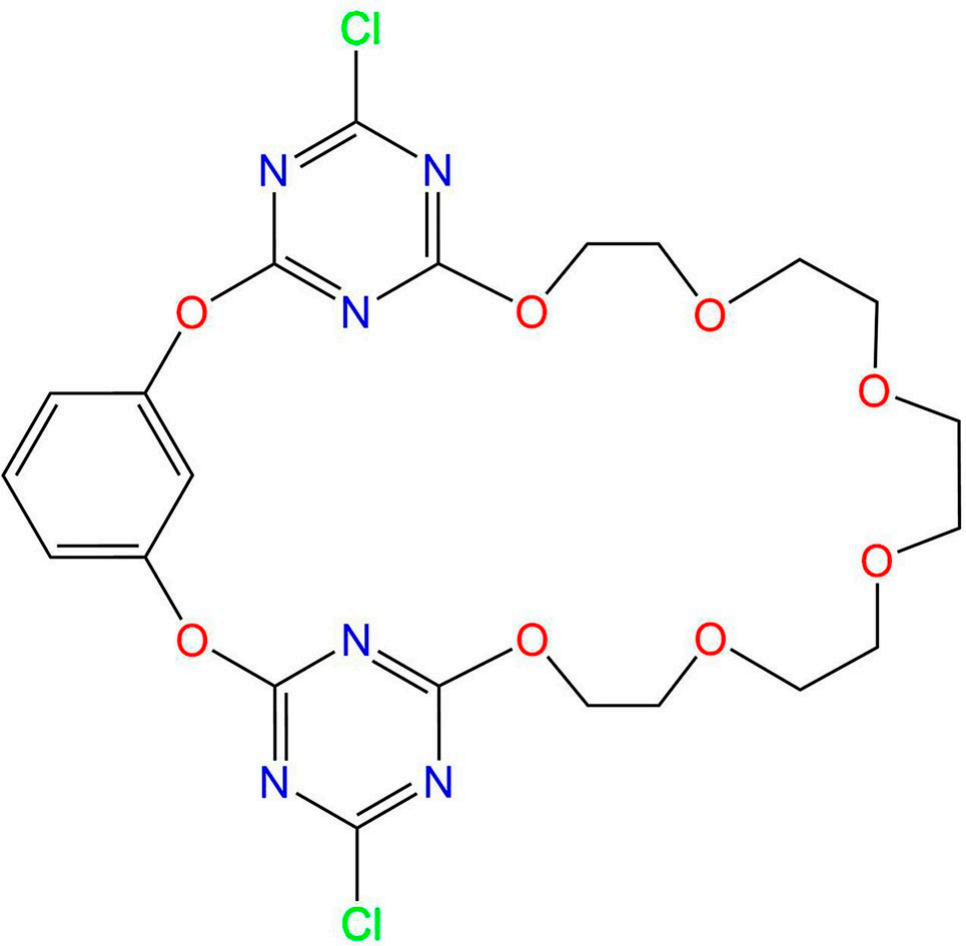
Structure of the dichlorooxacalix[2]arene[2]triazine‐related ion‐pair receptor **1**.^42^

To address this question we focus in the present study on a tritopic IP receptor, for which cation binding can be rationally designed using appropriately sized pentaethylene glycol chains,^1^, ^2^, ^3^, ^7^, ^8^, ^9^, ^10^, ^11^ while anion coordination can be achieved by interaction of anions with electron‐deficient aromatic triazine rings.^51^, ^52^, ^53^, ^54^, ^55^, ^56^, ^57^, ^58^, ^59^, ^60^, ^61^, ^62^, ^63^ The bridging oxygen atoms conjugate with the triazines such that the aromatic trimeric fragment tends to form a pre‐organized V‐shaped pocket in which two triazines serve as homoditopic binding sites for anions.^59^, ^61^ Receptor **1** 42 (Scheme 1) is based on a triazine‐containing aromatic trimer fragment for anion recognition and a pentaethylene glycol chain for cation chelation. **1** has been reported to form stable complexes with Ca^2+^ salts but shows low affinity to Mg^2+^.^42^

Here we report on ion pairing of Sr(ClO_4_)_2_, Ba(ClO_4_)_2_, and SrI_2_ dissolved in acetonitrile in the presence and absence of receptor **1**. To explore to what extent **1** can induce ion pairing and the corresponding structures of the IPs formed, we use a combination of experiments. To quantify ion association in solution, we use dielectric relaxation spectroscopy (DRS), which is sensitive to the rotation of dipolar species and as such can detect both IPs bound by the molecular scaffold of the receptor and bare IPs. We compare these results to those obtained from ^1^H nuclear magnetic resonance spectroscopy (^1^H NMR) titrations. To obtain information on the composition and structure of the formed complexes, we use electrospray ionization mass spectrometry (ESI‐MS), single crystal X‐ray diffraction (XRD), and density functional theory (DFT) calculations. We find that all salts have a marked tendency to form IPs in acetonitrile in the absence of **1**. The addition of **1**, which in solution adopts an open form favorable for ion binding, stabilizes IPs and thus results in the enhancement of ion association.

## Experimental Section

### Sample Preparation

Samples were prepared using HPLC grade acetonitrile (Fisher Scientific), deuterated acetonitrile, or HPLC‐MS grade acetonitrile (VWR Chemicals) as solvents. Salts for DRS and ESI‐MS experiments SrI_2_ (Alfa Aesar, 99.99 %), Sr(ClO_4_)_2_ ⋅ 3H_2_O (Alfa Aesar, 98 %), BaI_2_ (Alfa Aesar, 99.99 %) and Ba(ClO_4_)_2_ (Acros Organics, 99 %) were used without further purification. To avoid the contribution of water to the dielectric spectra, Sr(ClO_4_)_2_ ⋅ 3H_2_O was dried in vacuo at 160–170 °C until constant weight was reached. For ^1^H nuclear magnetic resonance spectroscopic (NMR) titrations, all metal (Sr(ClO_4_)_2_ ⋅ 6H_2_O, Ba(ClO_4_)_2_ ⋅ 3H_2_O) and tetrabutylammonium salts (Bu_4_NCl, Bu_4_NBr and Bu_4_NI) were used as received. Receptor **1** was synthesized according to the procedure reported in Ref. [42].

### Dielectric Relaxation Spectroscopy (DRS)

DRS probes the frequency‐dependent macroscopic polarization of a sample in an external electric field^64^−^66^ with field frequency *ν*, which is generally expressed in terms of the complex permittivity, $\hat{\epsilon }\left(\nu \right)$ :

(1) $$\;\hat{\epsilon }\left(\nu \right)=\epsilon {}^{'}\left(\nu \right)-\epsilon {}^{'}{}^{'}\left(\nu \right)-\frac{i\kappa }{2\pi \nu \epsilon_{0}}$$

with *ϵ’*(*ν*) and *ϵ”*(*ν*) being the frequency‐dependent dielectric permittivity and loss, respectively; and *ϵ*_0_ is the permittivity of free space. For conducting samples, the translational motion of mobile ions gives rise to Ohmic loss (last term of Eq. 1), which scales with the conductivity, *κ*, of the sample. We assume *κ* to be real and independent of *ν* (i. e. the dc conductivity).

At microwave frequencies, polarization stems predominantly from rotation of species with an electrical dipole moment. Thus, besides its sensitivity to dipolar molecules, DRS is particularly sensitive to the formation of IPs in solution, as the oppositely charged ions of an IP are separated by a well‐defined separation distance, yielding an intrinsically high dipole moment. As the dipole moment increases with increasing distance between cation and anion, DRS can distinguish between different IP species, like contact or solvent‐separated ion‐pairs.^65^, ^66^ For any dipolar relaxation (e. g. solvent or IPs), a dispersion in the real part, *ϵ’*(*ν*), and a peak in the imaginary part, *ϵ”*(*ν*), are observed.

The $\hat{\epsilon }$ (*ν*) spectra were recorded at room temperature ((23±2) °C), using an Anritsu Vector Network Analyzer (model MS4647 A). The frequency range at 0.2≤*ν*/GHz≤50 was covered using a frequency‐domain reflectometer, equipped with a coaxial open‐ended probe based on 1.85 mm connectors. Spectra at 60≤*ν*/GHz≤125 were recorded using an open‐ended probe connected with 1 mm connectors to an external frequency converter module (Anritsu 3744 A mmW).^67^ To calibrate the setup, air, conductive silver paint, and acetonitrile^68^ were used as calibration standards.

To study solutions of the salts in acetonitrile using DRS, samples with *c_salt_* up to 0.14–0.16 M were prepared. To study the binding of salts to **1** in acetonitrile, two series of solutions were prepared. First, *c*_**1**_ was varied from 0 to 0.11 M at a constant *c_salt_* (0.10 M), except for BaI_2_, which is not sufficiently soluble in acetonitrile. Second, *c_salt_* was increased from 0 to 0.14 M at constant *c*_**1**_ (0.05 M). All solution compositions are listed in Tables S1 and S2 in the Supporting Information (SI).

### ^1^H Nuclear Magnetic Resonance (NMR) Spectroscopy

To supplement the quantitative findings from the DR spectroscopic measurements, we performed ^1^H NMR titrations. The data evaluation was performed with the *PSEQUAD* 69 and *Bindfit* 70 software packages. More experimental details are given in the SI.

### Single‐Crystal X‐ray Diffraction

Mixtures of **1** and M(ClO_4_)_2_ (M=Ba, Sr) were dissolved in CH_3_OH/CHCl_3_, and ethyl ether was allowed to slowly diffuse into the solution at 273 K to produce single crystals for X‐ray analysis. Single crystal X‐ray diffraction data were collected on a MM007HF Saturn724+ diffractometer using MoK/α radiation (*λ*=0.71073 Å) at a temperature of 173 K. The intensity data were collected by the omega scans techniques, scaled, and reduced with the *CrystalClear* software.^71^ X‐rays were provided by a fine‐focus sealed X‐ray tube operated at 50 kV and 24 mA. Integrated reflection intensities were produced and the correction of the collected intensities for absorption was done using *CrystalClear*. The structures were solved by direct methods using *SHELXT* 72 and refined using full‐matrix least‐squares methods implemented in the *SHELXL* 73 program. All non‐hydrogen atoms were refined anisotropically, and hydrogen atoms attached to carbon atoms were fixed at their ideal positions.

### Electrospray Ionization Mass Spectrometry

To assess the composition of ionic/molecular complexes in solution, electrospray ionization (ESI) mass spectra were recorded using an Advion Expression‐L Compact Mass Spectrometer equipped with a single quadrupole separator, providing an average resolution of 0.5 *m*/*z* units. Here, the range of 100≤*m*/*z*≤1200 was scanned in the positive‐ion mode. Experiments were performed using samples with *c_salt_*=*c*_**1**_=0.01 M (SrI_2_, Sr(ClO_4_)_2_, BaI_2_, Ba(ClO_4_)_2_) or *c_salt_*=*c*_**1**_=0.02 M (SrI_2_).

### Density Functional Theory (DFT) Calculations

The geometries of **1** and the ion‐pair complexes at various configurations were optimized with the *Gaussian* 09^74^ software using the B3LYP hybrid DFT functional^75^, ^76^ and the def2‐TZVP or its def2‐TZVPD variant including diffuse functions^77^ to take noncovalent interactions into account. For all calculations, Grimme's D3 empirical dispersion correction^78^ was employed. Implicit solvent effects were taken into account applying the conductor‐like polarizable continuum model (CPCM)^79^ with acetonitrile as solvent.

## 2 Results and Discussion

### 2.1 Ion Pairing in the Absence of the Receptor

**Figure 1:**
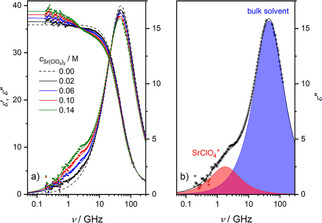
(a) Relative permittivity (*ϵ’*, triangles, left axis) and dielectric loss (*ϵ”*, squares, right axis) spectra of 0–0.14 M Sr(ClO_4_)_2_ solutions. Solid lines are the results of fitting Eq. 2 to the data; dashed line shows the spectrum of pure acetonitrile, taken from Ref. [68]. (b) Contribution of acetonitrile (blue‐shaded area) and SrClO_4_^+^ ion‐pairs (red‐shaded area) to *ϵ”* for 0.14 M Sr(ClO_4_)_2_, as obtained from the fit. Symbols represent the experimental data, and the black solid line is the result of the fit. In both panels, the last term of Eq. 2 has been subtracted from *ϵ”* for visual clarity.

To explore ion pairing of the studied salts in the absence of the receptor molecules, we study the dielectric relaxation of solutions of Sr(ClO_4_)_2_ (Figure 1a), SrI_2_, and Ba(ClO_4_)_2_ (Figures S1a and S2a, SI) in acetonitrile. For all samples we observe a relaxation at ∼50 GHz, evidenced by a peak in the dielectric loss and a dispersion in the dielectric permittivity spectra (Figure 1), due to the solvent acetonitrile.^68^

As can be seen for Sr(ClO_4_)_2_ in Figure 1a (also for SrI_2_ and Ba(ClO_4_)_2_, see Figures S1a and S2a in the SI), upon dissolution of salt a shoulder in the imaginary part at ∼1 GHz emerges with increasing *c_salt_*, indicative of the formation of dipolar IPs.^65^, ^66^ Due to this low‐frequency relaxation, which also goes along with a dispersion in *ϵ’*(*ν*), the static permittivity (*ϵ_s_*, the low‐frequency plateau of *ϵ’*(*ν*)) exhibits an increase with increasing salt concentration. As such, the increase in sample polarization due to the formation of dipolar solute species, i. e. IPs, overcompensates its decrease due to the dilution of dipolar solvent molecules. This suggests that the studied iodide and perchlorate salts do not fully dissociate in acetonitrile due to its lower solvent permittivity and weaker solvation,^50^ giving rise to the formation of dipolar IPs (i. e., SrI^+^, SrClO_4_^+^, and BaClO_4_^+^).

To analyze the spectra quantitatively, we fit a relaxation model to the data. For the present samples we find that a combination of two Debye‐type relaxations^64^ accounting for the solvent (AN) and the ion‐pair (MX^+^) relaxations, respectively, provides an excellent description of the experimental spectra with the least number of adjustable parameters:

(2) $$\hat{\epsilon }\left(\nu \right)=\frac{S_{{MX}^{+}}}{1+i2\pi \nu \tau_{{MX}^{+}}}+\frac{S_{AN}}{1+i2\pi \nu \tau_{AN}}+\epsilon_{\infty }-\frac{i\kappa }{2\pi \nu \epsilon_{0}}$$

where *S*_MX+_ and *S*_AN_ are the MX^+^ and the solvent relaxation amplitudes, respectively, while *τ*_MX+_ and *τ*_AN_ represent the corresponding relaxation times. The infinite‐frequency permittivity, *ϵ*_∞_, comprises all contributions at frequencies higher than covered in our experiment. Such decomposed dielectric loss spectra of the 0.14 M salt solutions are depicted in Figures 1b, S1b, and S2b (see SI).

To obtain quantitative information about the degree of ion pairing, we use the Cavell equation,^65^, ^66^, ^80^ which relates *S*_MX+_ to the equilibrium concentration ([MX^+^]) and effective dipole moment (*μ*_MX+_) of the MX^+^ ion‐pairs:

(3) $$S_{{MX}^{+}}=\frac{\epsilon_{s}}{\epsilon_{s}+A_{{MX}^{+}}\left(1-\epsilon_{s}\right)}\;\cdot \;\frac{N_{A}}{{3k}_{B}T\epsilon_{0}}\;\cdot \;\left[{MX}^{+}\right]\;\cdot \;{\mu_{{MX}^{+}}}^{2}$$

where *A*_MX+_ is the so‐called cavity‐field factor (determined by the geometry of the rotating particle), *N_A_* is the Avogadro number, *k_B_* is the Boltzmann constant, and *T* is the thermodynamic temperature.

In order to calculate [MX^+^] from *S*_MX+_, the value of *μ*_MX+_, which predominantly depends on the spatial separation between cation and anion, needs to be known. In salt solutions, both contact (CIP, direct contact between the cation and anion) and solvent‐shared (SIP, cation and anion are separated by one solvent molecule) IPs are conceivable species.^50^, ^65^, ^66^ Despite also the existence of triple IPs (consisting of one cation and two anions or one anion and two cations) has been inferred from infrared spectra for Mg(ClO_4_)_2_ and Ca(ClO_4_)_2_ in acetonitrile, only one dipolar relaxation mode has been detected in the DRS spectra for these salts,^68^ in line with our present findings. Given the low salt concentrations of the present samples, at which triple ion aggregates are minor species also for Mg(ClO_4_)_2_ and Ca(ClO_4_)_2_, we ascribe the low‐frequency relaxation mode to CIPs or SIPs.

**Figure 2:**
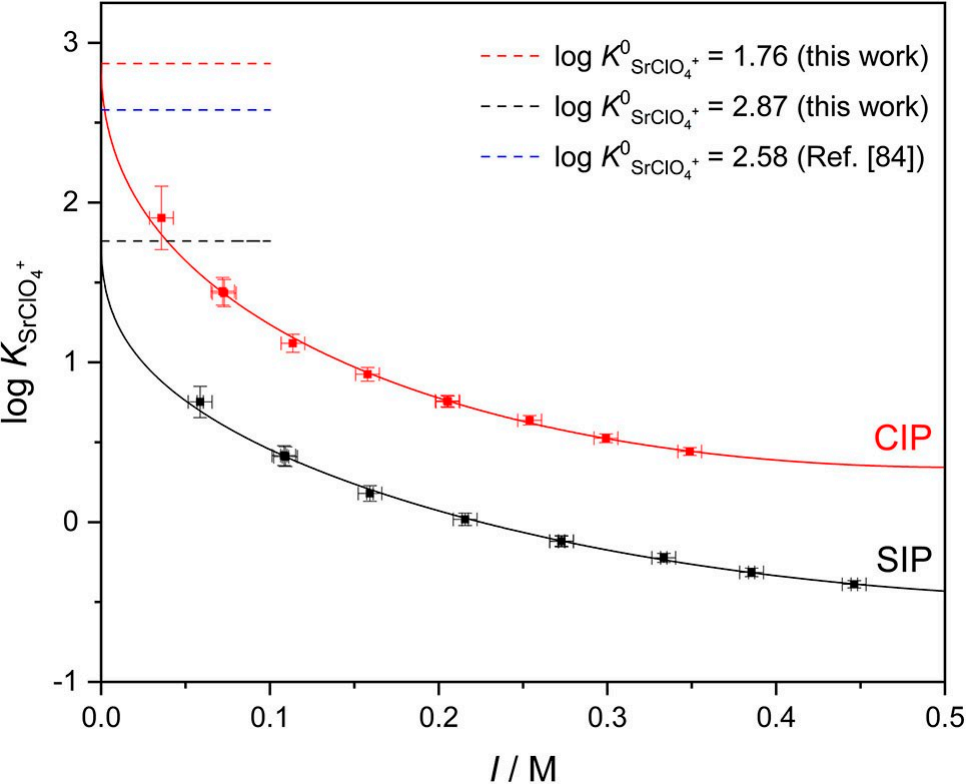
Experimental ion‐pair formation constants, log *K*_SrClO4+_, assuming the formation of contact (CIP, red symbols) or solvent‐separated (SIP, black symbols) SrClO_4_^+^ ion‐pairs as a function of ionic strength (*I*). Solid lines show fits using Eq. 5; error bars were calculated assuming *σ*(*S*_MX+_)=±0.3. Dashed lines indicate the thermodynamic formation constants (log *K* ^0^_SrClO4+_) at infinite dilution, obtained in this work or reported in Ref. [84].

To determine which IP species prevails in solution, we consider the two limiting cases: exclusive formation of either CIPs or SIPs. Based on the geometric model described in detail in Ref. [80], we calculate *μ*_CIP_, *μ*_SIP_, *A*_CIP,_ and *A*_SIP_ (Table S3, SI) using data for ionic radii, solvent radii, and polarizabilities from Refs. [81–83]. From these values, we obtain [MX^+^] using Eq. 3. Assuming that [MX^+^]=[CIP] or [SIP], we calculate the IP formation constants, *K*_MX+_, for the CIP (*K*_CIP_) and SIP (*K*_SIP_) species via Eq. (4):

(4) $$K_{{MX}^{+}}=\frac{\left[{MX}^{+}\right]\;\cdot \;c^{⊘}}{\left[M^{+}\right]\;\cdot \;\left[X^{-}\right]}=\frac{\left[{MX}^{+}\right]{\;\cdot \;c}^{⊘}}{(c_{salt}-[{MX}^{+}])\;\cdot \;(2c_{salt}-[{MX}^{+}])}$$

where [M^+^] and [X^−^] are the free cation and anion concentrations and *c* ^ø^ the standard molar concentration (1 M). As shown in Figure 2, the equilibrium constants for the two limiting cases (SIP and CIP) decrease with increasing salt concentration due to increased charge screening.^50^ Yet the curves are offset as a result of the different absolute values of $\mu_{MX+}$ for the CIP and SIP species. To elucidate which IP species predominates association equilibria, the formation constants have to be compared to literature data obtained from independent experimental techniques.

**Figure 3:**
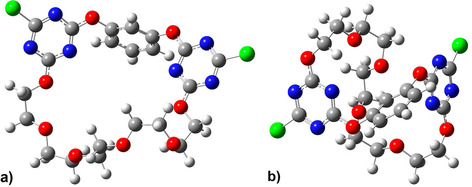
Structures of the open (a) and twisted (b) conformers of receptor **1**, optimized at the B3LYP‐D3/def2‐TZVP level. Implicit solvent effects were taken into account applying the CPCM approach. The calculated effective dipole moments are 8.3 D (a) and 1.6 D (b).

To exclude differences arising from different experimental sensitivities and ionic strengths, such comparison should be based on the standard thermodynamic association constant, *K* ^0^_MX+_ (i. e. the limiting value of *K*_MX+_ at infinite dilution). To obtain *K* ^0^_MX+_, we extrapolate the values of *K*_MX+_ to zero ionic strength using a Guggenheim‐type equation:^65^, ^68^

(5) $$\log K_{{\mathrm{MX}}^{+}}=\log{K^{0}}_{{MX}^{+}}-\frac{2A_{DH}\left|\left.z_{+}z_{-}\right|\right.\sqrt{I}}{1+B_{DH}d\sqrt{I}}+CI+DI^{1.5}$$

where *A*_DH_ and *B*_DH_ are the Debye‐Hückel constants at *T*=23 °C,^68^ *d* is the distance of charge separation, and *C* and *D* are adjustable parameters. We calculate the ionic strength, *I*, from *c_salt_* by correcting for the IPs formed (that is, *I*=3*c_salt_*–2[MX^+^]) and fit Eq. 5 to the data in Figure 3. Error bars (±*σ*) for log *K*_MX+_ and *I* were estimated assuming *σ*(*S*_MX+_)=0.3. The fitted parameters of Eq. 5 for each IP are listed in Table S4, SI.

Figure 2 demonstrates that for SrClO_4_^+^ (and also for SrI^+^ and BaClO_4_^+^, see Figures S3 and S4 in the SI), Eq. 5 describes the ionic strength dependence for both constants, *K*_CIP_ and *K*_SIP_, very well. This comparison shows that the extrapolated values (marked as dashed lines in Figure 2) agree well with those derived from previous conductometric experiments for the same salts,^84^ if we assume the exclusive formation of CIPs (see also Table 1). Hence, this agreement provides evidence for CIP being the dominant ion‐pair species for the studied salts in acetonitrile.

**Table 1:** Formation constants (log *K* ^0^_MX+_±*σ* at (23±2) °C) of MX^+^ ion‐pairs, assuming contact (CIP) or solvent‐separated ion‐pairs (SIP) as obtained from the dielectric relaxation amplitudes. Also listed are the values from Ref. [84] (25 °C) determined using conductometry. The last column lists the degree of ion pairing as obtained in the present study at *c_salt_*=0.1 M.

Reaction	SIP	CIP	Ref. [84]	% [MX^+^]/*c_salt_*
Sr^2+^+I^−^ $\vec{\leftarrow }\;$ SrI^+^	1.95±0.03	3.39±0.13		53
Sr^2+^+ClO_4_^−^ $\vec{\leftarrow }\;$ SrClO_4_^+^	1.76±0.03	2.87±0.04	2.58±0.01	46
Ba^2+^+ClO_4_^−^ $\vec{\leftarrow }\;$ BaClO_4_^+^	1.72±0.04	2.67±0.04	2.69±0.02	43

We note that this notion partially contrasts earlier findings for solutions of Mg(ClO_4_)_2_ and Ca(ClO_4_)_2_ in acetonitrile, where – despite CIPs also dominate – the formation of both CIP and SIP at *c_salt_*<0.2 M has been suggested.^68^ This apparent discrepancy can be rationalized on the basis of different ionic radii: smaller ionic radii give rise to higher ionic surface charge density and thus to stronger solvation. As such, the interaction of acetonitrile with the smaller Mg^2+^/Ca^2+^ cations is stronger than with the larger ions Sr^2+^ and Ba^2+^. In turn, weaker solvation of Sr^2+^ and Ba^2+^ relative to Mg^2+^ and Ca^2+^, likely makes SIPs less significant for the salts studied in this work.

Overall, our findings for these solutions imply that despite perchlorate is often considered as weakly coordinating anion, we find that both Sr(ClO_4_)_2_ and Ba(ClO_4_)_2_ tend to form IPs in acetonitrile, in line with previous studies.^68^, ^84^ The fraction of ions that form IPs (% [MX^+^]/*c_salt_* at *c_salt_*=0.1 M, Table 1) exceeds 40 % for the perchlorates, while for SrI_2_ our results suggest that more than 50 % of all ions are bound in CIPs. In turn, only a fraction of ions is present in solution as free ions. Upon addition of IP receptors, which will be discussed below, binding of both, free ions and ion‐pairs to the receptor can occur.

### 2.2 The Structure of Receptor 1 in Acetonitrile

Before discussion of binding of salts to the receptor, we first investigate the structure of receptor **1** in solution. Crystallographic experiments have shown that **1** exists in two markedly different conformations in the solid state,^42^ here referred to as ‘open’ and ‘twisted’ conformer (Figure 3). Given the different symmetries of both conformers, studying the dielectric relaxation of **1** can provide information on the most stable conformation in acetonitrile.

For solutions of 0.05 M of **1**, we detect a small‐amplitude relaxation at ca. 1 GHz in the DR spectrum (amplitude *S*_**1**_≈0.3, relaxation time *τ*_**1**_≈130 ps, see the decomposed loss spectrum in Figure S5, SI). Based on Eq. 3, this relaxation amplitude corresponds to a dipolar species with a dipole moment of *μ*_**1**_=(8.3±0.8) D. We compare this value with the structures of the open and twisted conformers (Figure 3), obtained from geometry optimizations at the B3LYP‐D3/def2‐TZVP level of theory, taking implicit solvent effects into account. These calculations suggest that the calculated dipole moment of the open form is *μ_calc_*=8.3 D, which is in excellent agreement with the experimental value. Conversely, due to its high symmetry, the twisted conformer has a much lower dipole moment (*μ_calc_*=1.6 D). Thus, our results indicate that in solution the open conformer prevails. In this geometry the ion binding sites are pre‐organized such that both cations and anions can readily access the binding pockets, in contrast to the twisted form.

### 2.3 Ion Pairing in the Presence of the Receptor

#### 2.3.1 Qualitative Findings for the Binding of Salts Both in the Solid and Solution Phases

**Figure 4:**
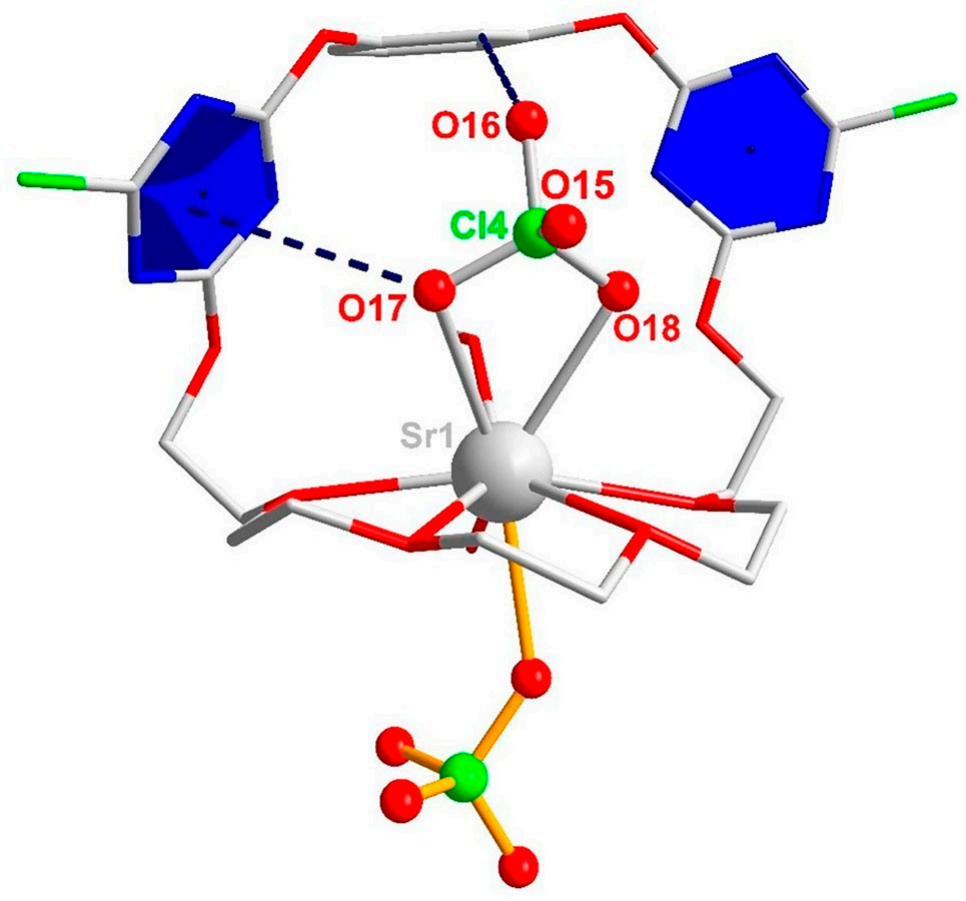
Crystal structure of the [**1** ⋅ Sr(ClO_4_)_2_] ⋅ H_2_O ⋅ CH_3_OH ion‐pair complex. The anion‐π distance is indicated by a dashed line between the O17 atom and the blue triazine plane (*d*_O17–plane_=3.393 Å). Also shown is the intramolecular H bond between the H^*f*^ aryl proton and O16 (*d*_O16⋅⋅⋅H*f*_=2.486 Å).

Having established the relaxation dynamics of the binary solutions, we now turn to ternary samples where both receptor **1** and salt are present. Through the diffusion of ethyl ether to a mixture of receptor and alkali earth metal salts in CH_3_OH/CHCl_3_, we obtained single crystals of the [**1** ⋅ Sr(ClO_4_)_2_] ⋅ H_2_O ⋅ CH_3_OH (Figure 4 and Table S5, SI) and [**1** ⋅ Ba(ClO_4_)_2_] ⋅ 2H_2_O (Figure S6 and Table S6, SI) complexes. From the crystal structures, we find the cation is coordinated equatorially by the oxygens of the glycol chain, and are axially coordinated by the anion. The respective coordination number (CN) is 9 for Sr^2+^ and 10 for Ba^2+^; the higher CN of the latter reflects its higher ionic radius, providing room for more ligands.

In the structure of **1** ⋅ Sr(ClO_4_)_2_ (Figure 4), one ClO_4_^−^ is located outside the receptor, interacting electrostatically with the cation. The second anion resides in the aromatic binding pocket, being stabilized by a triazine moiety through anion‐π interaction, as indicated by the short distance between the aromatic ring and the O17 atom of the anion (3.393 Å). Moreover, an additional hydrogen bond between the O16 atom of ClO_4_^−^ and the H^*f*^ proton of the central aromatic ring is formed. Based on the distance (*d*_O16⋅⋅⋅H*f*_=2.486 Å), this hydrogen bond can be considered weak.^85^, ^86^

The crystal structure of the **1** ⋅ Ba(ClO_4_)_2_ IP complex shows similar binding motifs, with each Ba^2+^ coordinated by ten oxygens, four from the glycol chain, one from a water molecule and five from three ClO_4_^−^ anions (Figure S6, SI). The perchlorates are additionally coordinated by triazine rings, evidenced by the short distances between the O15, O16 atoms and the triazine planes (*d*_O ‐plane_=2.944 and 3.063 Å). The perchlorates also act as bridge to link two IP complexes to form a dimer.

To obtain information on the binding of salts to **1** in the solution phase, we carried out ESI‐MS measurements in solutions with *c_salt_*=*c*_**1**_=0.01 or 0.02 M. In the positive‐ion mode, we observe peaks due to **1** ⋅ SrI^+^, **1** ⋅ SrClO_4_^+^, **1** ⋅ BaI^+^ and **1** ⋅ BaClO_4_^+^ as well as to the bare and solvated **1** ⋅ Sr^2+^ and **1** ⋅ Ba^2+^ complexes (Figures S7–S11, SI). This suggests that both, CIPs and cations bound to **1**, coexist in solution. Based on the relative peak intensities, the **1** ⋅ MX^+^ complexes prevail at 1 : 1 and higher *c*_**1**_:*c_salt_* ratios (except for BaI_2_, see Figure S10, SI). Conversely, peaks corresponding to the free IPs are absent for all salts. Despite one cannot fully exclude their formation based solely on the mass spectra, their absence suggests that dissociation of **1** ⋅ MX^+^ is energetically more demanding than dissociation of MX^+^. Thus, **1** ⋅ MX^+^ complexes are the dominant ion‐pair species in the presence of the receptor.

**Figure 5:**
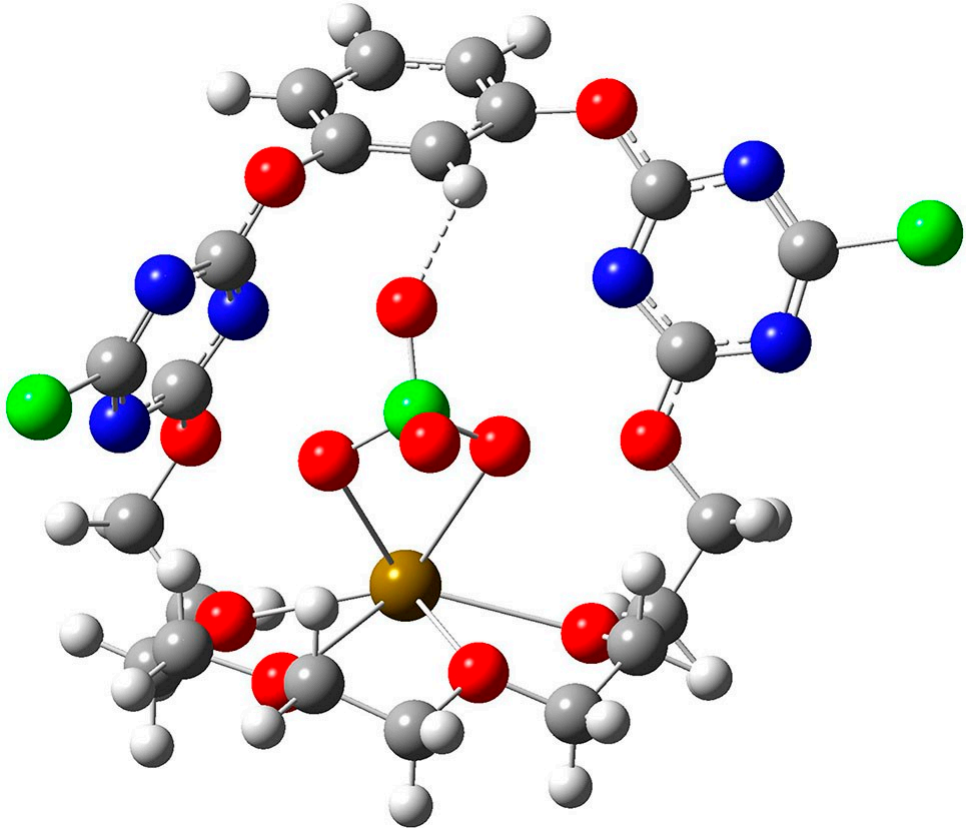
Structure of the SrClO_4_^+^ ion‐pair bound to receptor **1**, optimized at the

To gain further insights into the structure of the receptor‐bound IPs, we optimized the geometry of the **1** ⋅ SrClO_4_^+^, **1** ⋅ BaClO_4_^+^, **1** ⋅ SrI^+^ and **1** ⋅ BaI^+^ species at the B3LYP‐D3/def2‐TZVPD level of theory (Figure 5 as well as Figures S12–S14, SI), as these species dominate DRS relaxations at *c*_**1**_:*c_salt_*≈1 : 1 ratio (see below). In the perchlorate species the anion forms a hydrogen bond to the aryl proton (H^*f*^). The bond lengths are 2.520 Å (**1** ⋅ BaClO_4_^+^) and 2.419 Å (**1** ⋅ SrClO_4_^+^), with the latter agreeing well with the one found in the **1** ⋅ Sr(ClO_4_)_2_ crystal. Together with the C−H⋅⋅⋅O bond angles (139.0° and 149.7°, respectively), these characteristics are common for weak C−H⋅⋅⋅O hydrogen bonds.^85^, ^86^ As for the iodide complexes, we find strong interaction between I^−^ and the same aryl proton. The calculated distances (**1** ⋅ SrI^+^: 3.382 Å, **1** ⋅ BaI^+^: 3.163 Å) indicate the formation of a strong C−H⋅⋅⋅I^−^ bond.^87^ From these findings we conclude that in addition to anion‐π interaction, an intramolecular C−H⋅⋅⋅X^−^ hydrogen bond also contributes to the stabilization of the receptor‐bound anion.

#### 2.3.2 Quantifying Ion Pairing in the Presence of 1 by DRS

**Figure 6:**
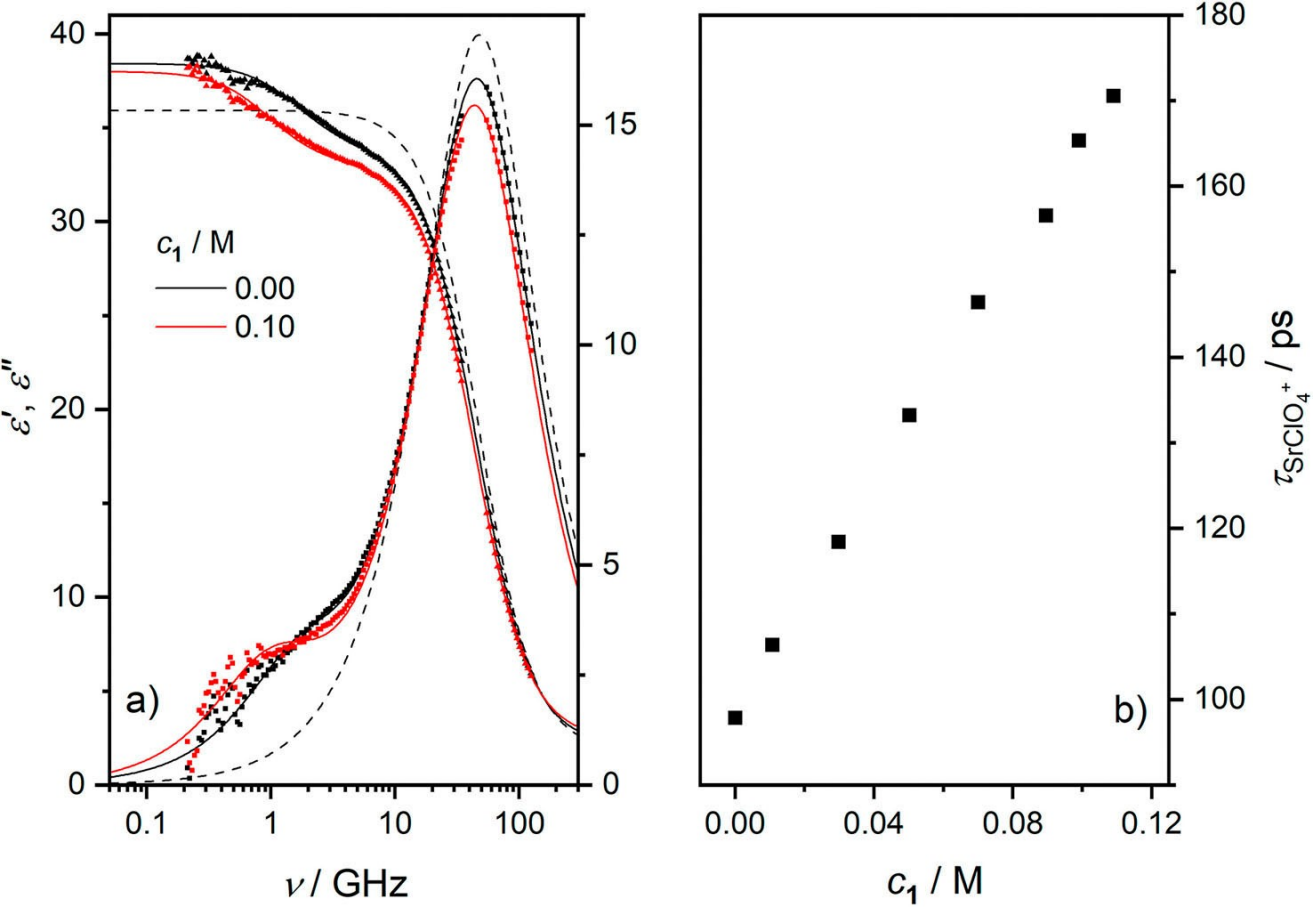
(a) Relative permittivity (*ϵ’*, triangles, left axis) and dielectric loss (*ϵ”*, squares, right axis) spectra for a 0.10 M Sr(ClO_4_)_2_ solution with (red symbols) and without (black symbols) receptor **1**. Solid lines are the results of fitting Eq. 2 to the data; dashed line shows the spectrum of pure acetonitrile, taken from Ref. [68]. The last term of Eq. 2 has been subtracted from *ϵ”* for visual clarity. (b) Ion‐pair relaxation time in 0.10 M Sr(ClO_4_)_2_ as a function of receptor concentration.

To study the formation of IPs in the presence of **1**, we recorded dielectric spectra of the ternary samples. Upon addition of **1** to solutions of 0.10 M Sr(ClO_4_)_2_, SrI_2_ and Ba(ClO_4_)_2_ (Figure 6a as well as Figures S15a–17a, SI), we find a shift of the IP relaxation, i. e. the dispersion in *ϵ’* and the shoulder in *ϵ”* at ∼1 GHz, to lower frequencies. The relaxation amplitude increases slightly upon addition of **1** (see also discussion below). The small variation of the IP amplitude directly implies that the spatial separation of the underlying dipolar species is only little affected by the presence of **1**, given that the overall concentration of IPs does not decrease in the presence of **1** (see Eq. 3). The decrease of the solvent relaxation amplitude is due to the reduced concentration of the solvent, but its peak position (∼50 GHz=(2*πτ*_AN_)^−1^) in the dielectric loss spectrum is hardly affected by the presence of the receptor.

Also for the ternary samples Eq. 2 describes the experimental spectra well; the contributions of both relaxations to the overall loss spectra for solutions *c_salt_*=*c*_**1**_=0.10 M are plotted in Figures 15b–17b in the SI. The presence of only two discernible relaxations (solvent and ion‐pairs) implies that we cannot resolve separate relaxations due to receptor‐bound salts and due to bare IPs. Yet, the variation of the peak position of the solute mode – in contrast to the solvent peak – indicates the binding of MX_2_ salts (or IPs) to **1**: we find the extracted values of the IP relaxation time, *τ*_MX+_, to increase with increasing concentration of **1 (**Figure 6b and S18, SI). For diffusive rotation, *τ*_MX+_ is proportional to the hydrodynamic volume of the rotating species and to the viscosity of the sample.^88^ The low concentration of solutes and the insensitivity of *τ*_AN_ to the addition of **1** renders increasing viscosity unlikely. Rather, the large increase of *τ*_MX+_ provides evidence for the formation of receptor‐bound complexes: the **1** ⋅ MX^+^ species have a larger volume than the bare MX^+^ IPs. As such, the DRS relaxation times indicate that for all studied salts, **1** ⋅ MX^+^ is the major IP species at high concentrations of **1**, in line with the ESI‐MS spectra. This notion is further supported by ^1^H NMR titration experiments, which indicate that **1** ⋅ MX_2_ complexes are only significant for an excess of iodide (see discussion in the SI together with Figures S19–S24 and Table S7).

To relate the relaxation amplitudes, *S*_MX+_, to IP concentrations, the contribution of both MX^+^ and **1** ⋅ MX^+^ species to *S*_MX+_ has to be taken into account. For quantitative analysis of ion pairing using Eq. 3, the dipole moment of **1** ⋅ MX^+^, *μ*_**1** ⋅ MX+_, is required. Based on the direct contact between the cation and the anion in the crystal structure (Figures 4 and S6, SI), one might expect *μ*_**1** ⋅ MX+_≈*μ*_MX+_, which is confirmed by DFT calculations that show the dipole moments of the receptor‐bound and bare IPs to agree within less than 3 D (see Table S3, SI). Experiments on solutions of BaI_2_+**1** further support the similar dipole moments of **1** ⋅ MX^+^ and MX^+^ (see the discussion together with Figures S25–S27 in the SI).

**Figure 7:**
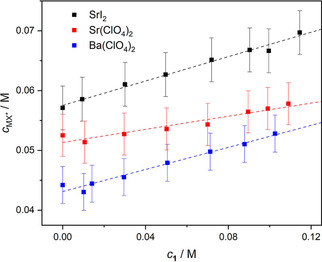
Concentrations of bare/receptor‐bound MX^+^ ion‐pairs for 0.10 M SrI_2_, Sr(ClO_4_)_2_ and Ba(ClO_4_)_2_ solutions as a function of concentration of **1**. The values of *c*_MX+_ and their error bars were calculated via Eq. 3, assuming *σ*(*S*_MX+_)=±0.3. The dashed lines are guide to the eye.

Thus, assuming *μ*_**1** ⋅ MX+_ ≈ *μ*_MX+_, the total IP concentration, *c*_MX+_ (≈[MX^+^]+[**1** ⋅ MX^+^]), in the presence of the receptor can be extracted from *S*_MX+_ using Eq. 3. The thus obtained values for *c*_MX+_ (Figure 7) show an increase in [MX^+^]+[**1** ⋅ MX^+^] by ∼10–25 %, upon addition of ∼0.1 M **1** to the 0.1 M salt solutions. Consequently, ion association is enhanced in the presence of **1**. This increase in ion pairing is consistent with the 15–25 % decrease of the conductivities (Figure S28, SI) of the samples as the **1** ⋅ MX^+^ complexes hardly contribute to the overall conductivity due to their reduced mobility or charge/volume ratio.^89^ We note that similar conclusions can be obtained from the experiments where *c_salt_* gradually increases (0.02–0.14 M) at *c*_**1**_=0.05 M: addition of **1** results in higher relaxation times, higher IP concentrations as well as lower conductivities (Figures S29–S34, SI).

Our results thus show that the presence of **1** enhances IP formation for all studied salts. For the overall degree of ion association (([MX^+^]+[**1** ⋅ MX^+^])/*c_salt_*), we find 67 % for SrI_2_, 57 % for Sr(ClO_4_)_2_ and 50 % for Ba(ClO_4_)_2_ in the presence of 0.10 M **1** (see Table 1). Overall, the trend in the ion association strength is the same (SrI_2_>Sr(ClO_4_)_2_>Ba(ClO_4_)_2_) with and without **1**. Assuming that the formation of **1** ⋅ MX^+^ prevails at *c*_**1**_/*c_salt_*≥**1** ratios (that is, *c*_**1** ⋅ MX+_+*c*_MX+_≈*c*_**1** ⋅ MX+_), we can estimate the cumulative stability constants of the **1** ⋅ MX^+^ IP complexes for samples containing 0.10 M **1**:

(6) $$K_{1\cdot {\mathrm{MX}}^{+}}=\frac{\left[1\cdot {\mathrm{MX}}^{+}\right]\cdot {\left(c^{\emptyset }\right)}^{2}}{\left[M^{2+}\right]\cdot \left[I^{-}\right]\cdot \left[1\right]}=\frac{\left[1\cdot {\mathrm{MX}}^{+}\right]\cdot {\left(c^{\emptyset }\right)}^{2}}{\left(c_{salt}-\left[1\cdot {\mathrm{MX}}^{+}\right]\right)\cdot \left(2c_{salt}-\left[1\cdot {\mathrm{MX}}^{+}\right]\right)\cdot \left(c_{1}-\left[1\cdot {\mathrm{MX}}^{+}\right]\right)}$$

The thus calculated constants are listed in Table 2. Accordingly, we find stronger association for Sr^2+^ salts as compared to Ba^2+^ showing that Sr^2+^ matches better the size of the polyethylene‐glycol binding cavity. Also, this trend is consistent with that of the cation‐binding constants derived from ^1^H NMR titrations using perchlorate salts (i. e. *K*_**1** ⋅ Sr2+_>*K*_**1** ⋅ Ba2+_, see Table S7 in the SI). These constants are however consistently higher than *K*_**1** ⋅ SrClO4+_ and *K*_**1** ⋅ BaClO4+,_ obtained from DRS (Table 2). This discrepancy can be rationalized by the notion that DRS is sensitive only to the formation of dipolar **1** ⋅ MX^+^ complexes, while NMR detects all species that contain a cation (i. e. **1** ⋅ M^2+^+**1** ⋅ MX^+^): ^1^H NMR chemical shifts are primarily sensitive to the coordination of cations, while the binding of ClO_4_^−^ does not alter the protons’ chemical environment. Consequently, NMR yields higher equilibrium concentrations and thus higher formation constants (for discussion, see the SI).

**Table 2:** Stability constants (log *K* ± *σ*, at (23±2) °C) corresponding to the reactions in the first column, obtained in this work via DR spectroscopic measurements at *I* ≈0.17–0.19 M.

Reaction	log *K*
**1**+Sr^2+^+I^−^ $\vec{\leftarrow }\;$ **1** ⋅ SrI^+^	2.66±0.04
**1**+SrI^+^ $\vec{\leftarrow }\;$ **1** ⋅ SrI^+^	1.67±0.04
**1**+Sr^2+^+ClO_4_^−^ $\vec{\leftarrow }\;$ **1** ⋅ SrClO_4_^+^	2.35±0.03
**1**+SrClO_4_^+^ $\vec{\leftarrow }\;$ **1** ⋅ SrClO_4_^+^	1.52±0.03
**1**+Ba^2+^+ClO_4_^−^ $\vec{\leftarrow }\;$ **1** ⋅ BaClO_4_^+^	2.19±0.03
**1**+BaClO_4_^+^ $\vec{\leftarrow }\;$ **1** ⋅ BaClO_4_^+^	1.44±0.03

Overall, the ion association equilibria in the ternary systems studied in the present work consists of ion pairing and the binding of free ions as well as IPs by the receptor. Although the formation of **1** ⋅ SrI^+^ species refers to thermodynamic equilibrium and therefore we cannot derive if such complexes are formed via the binding of free ions or IPs (or both), it is possible to compare the formation constants of these processes: we estimate the equilibrium constant for binding of IPs to **1** (*K’*_**1** ⋅ MX+_) using the values of *K*_**1** ⋅ MX+_ (referring to the binding of free ions, Eq. 6) and *K*_MX+_ (referring to ion pairing, Eq. (7):

(7) $$K_{1\cdot {\mathrm{MX}}^{+}}^{'}=\frac{\left[1\cdot {\mathrm{MX}}^{+}\right]\cdot c^{\emptyset }}{\left[1\right]\cdot \left[{\mathrm{MX}}^{+}\right]}=\frac{K_{1\cdot {\mathrm{MX}}^{+}}}{K_{{\mathrm{MX}}^{+}}}$$

For this estimation we calculate the values of log *K*_MX+_ at the same ionic strength at which we determined the log *K’*_**1** ⋅ MX+_ constants (*I*≈0.16–0.19 M) using Eq. 5. The results (Table 2) suggest that **1** is also somewhat more efficient in binding SrI^+^ IPs as compared to SrClO_4_^+^ and BaClO_4_^+^. This difference can be explained by the stronger binding of Sr^2+^ as compared to Ba^2+^ as well as by the formation of strong hydrogen bonds to iodide as compared to perchlorate, as inferred from ^1^H NMR titrations and DFT geometries, respectively.

## 3 Conclusions

In the absence of **1**, SrI_2_, Sr(ClO_4_)_2_ as well as Ba(ClO_4_)_2_ tend to form 1 : 1 contact ion‐pairs (CIPs) in acetonitrile to a significant extent. The degree of ion pairing can be as high as ∼50 % for the I^−^ and ∼40 % for the ClO_4_^−^ salts, already at low salt concentrations (<0.15 M). The neat receptor **1** exists predominantly in an open form in solution, to which a cation can bind without major structural reorganization.

In the presence of **1** we find simultaneous binding of cations and anions to the receptor. The ESI‐MS results indicate that the receptor‐bound CIP species, i. e. **1** ⋅ SrI^+^, **1** ⋅ SrClO_4_^+^ and **1** ⋅ BaClO_4_^+^ prevail in solution over SrI^+^, SrClO_4_^+^ and BaClO_4_^+^ IPs. The formation of these receptor‐bound IP complexes is confirmed by the increasing DRS relaxation times in the presence of **1**. Quantitative analysis of the DRS results shows the overall degree of ion association to increase by approximately 10–25 % in the presence of **1**, relative to the receptor‐free solutions. Despite the overall enhancement of ion pairing in solution by the receptor is only moderate, a large fraction of IPs is complexed by **1** in solution. Structural analysis of the complexes reveals that their higher stability (as compared to the bare IPs) can be traced to the formation of coordinative, anion‐π as well as hydrogen bonding interactions.

We find that the overall degree of salt binding by **1** is higher for I^−^ salts than for ClO_4_^−^ salts. The extent of ion association follows the order of **1** ⋅ SrI_2_>**1** ⋅ Sr(ClO_4_)_2_ >**1** ⋅ Ba(ClO_4_)_2_, which also resembles the ion association trends of the bare salt solutions. These paralleling trends can be explained by that the cation‐anion distance, i. e. the electrostatic interaction between the co‐bound ions, remaining essentially unaffected upon binding to **1**. This holds also for perchlorate salts, even though perchlorate is often considered as weakly coordinating anion. Generally, our study shows that ion‐pair recognition is intimately related to ion pairing in solution, thereby highlighting the importance of taking the latter equilibrium into account when studying salt binding in organic solvents.

## Conflict of interest

The authors declare no conflict of interest.

## Supporting information

As a service to our authors and readers, this journal provides supporting information supplied by the authors. Such materials are peer reviewed and may be re‐organized for online delivery, but are not copy‐edited or typeset. Technical support issues arising from supporting information (other than missing files) should be addressed to the authors.

Supplementary
