# Supplementary material for: Enhancement of Ion Pairing of Sr(II) and Ba(II) Salts by a Tritopic Ion‐Pair Receptor in Solution
Source: Chemphyschem. 2020 Aug 13;21(17):1957–65. doi: 10.1002/cphc.202000507 (PMC7540308; doi:10.1002/cphc.202000507)
Supplement: Supplementary file 1 — Supplementary [file CPHC-21-1957-s001.pdf]

ChemPhysChem

Supporting Information

Enhancement of Ion Pairing of Sr(II) and Ba(II) Salts by a Tritopic Ion-Pair Receptor in Solution

Bence Kutus, Jun Zhu, Jian Luo, Qi-Qiang Wang, Alexandru Lupan, Amr A. A. Attia, De-Xian Wang,* and Johannes Hunger*

SUPPORTING INFORMATION

Table of contents

Sample compositions for DRS measurements 2

Dielectric relaxation spectra of receptor-free salt solutions 3

Dipole moments and thermodynamic association constants of ion-pairs 4

Dielectric relaxation spectrum for a solution of receptor 1 5

Parameters of the crystal structures of the [1•$Sr(ClO_4)_2$] and [1•$Ba(ClO_4)_2$] complexes 6

ESI-MS spectra of receptor-containing salt solutions 8

DFT optimized structures for the receptor-bound ion-pairs 10

Dielectric relaxation spectra and relaxation times of receptor-containing salt solutions (varying receptor concentration) 12

Determination of the cation- and anion-binding constants via ^{1}H NMR titrations 14

Determination of the dipole moment of the 1•BaI^+ complex 19

Conductivities of receptor-containing salt solutions (varying receptor concentration) 21

Relaxation times, conductivities and ion-pair concentrations for receptor-containing salt solutions (varying salt concentration) 21

References 24

Sample compositions for DRS measurements

Table S1 Analytical concentrations of salts (in M) in solutions prepared for DRS measurements.

SrI_2	$Sr(ClO_4)_2$	$Ba(ClO_4)_2$
0.021	0.022	0.022
0.043	0.041	0.031
0.060	0.059	0.040
0.080	0.079	0.062
0.100	0.100	0.080
0.119	0.121	0.101
0.140	0.139	0.119
	0.161	0.139
		0.159

Table S2 Analytical concentrations (in M) of salts and receptor **1** in solutions prepared for DRS measurements.

DRS measurements at c_{salt} = 0.10 M			DRS measurements at c_1 = 0.050 M		
1+SrI_2	1+$Sr(ClO_4)_2$	1+$Ba(ClO_4)_2$	1+SrI_2	1+$Sr(ClO_4)_2$	1+BaI_2
0	0	0	0	0	0
0.010	0.011	0.010	0.019	0.021	0.012
0.030	0.030	0.014	0.039	0.040	0.017
0.050	0.050	0.030	0.059	0.062	0.020
0.072	0.070	0.051	0.080	0.080	0.024
0.090	0.090	0.071	0.099	0.100	0.031
0.100	0.099	0.088	0.119	0.119	0.039
0.115	0.109	0.103	0.142	0.140	0.049

Dielectric relaxation spectra of receptor-free salt solutions

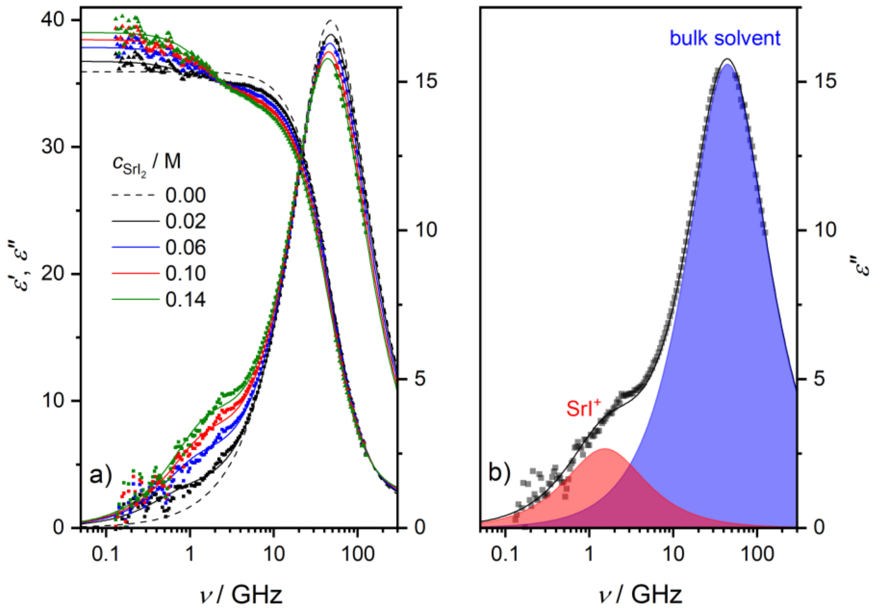

Figure S1 (a) Relative permittivity (ε', triangles, left axis) and dielectric loss (ε'', squares, right axis) spectra of 0–0.14 M SrI_2 solutions. Solid lines are the results of fitting Eq. 2 (main text) to the data; dashed line shows the spectrum of pure acetonitrile, taken from Ref. [S1]. (b) Contribution of acetonitrile (blue-shaded area) and SrI^+ ion-pairs (red-shaded area) to ε'' of the 0.14 M SrI_2 solution, as obtained from the fit. Squares represent the experimental data, and the black solid line is the result of the fit. In both panels, the last term of Eq. 2 has been subtracted from ε'' for visual clarity.

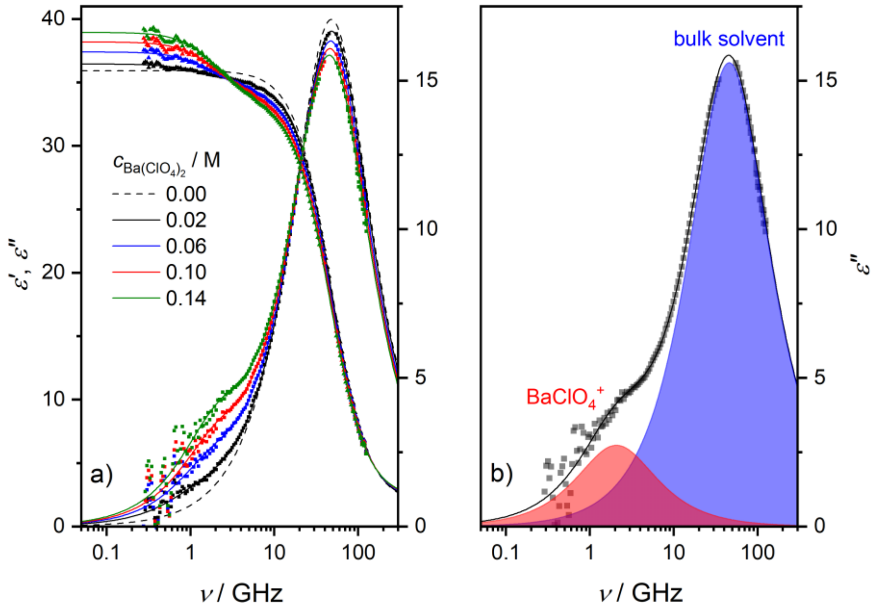

Figure S2 (a) Relative permittivity (ε', triangles, left axis) and dielectric loss (ε'', squares, right axis) spectra of 0–0.14 M $Ba(ClO_4)_2$ solutions. Solid lines are the results of fitting Eq. 2 (main text) to the data; dashed line shows the spectrum of pure acetonitrile, taken from Ref. [S1]. (b) Contribution of acetonitrile (blue-shaded area) and $BaClO_4^+$ ion-pairs (red-shaded area) to ε'' of the 0.14 M $Ba(ClO_4)_2$ solution, as obtained from the fit. Squares represent the experimental data, and the black solid line is the result of the fit. In both panels, the last term of Eq. 2 has been subtracted from ε'' for visual clarity.

Dipole moments and thermodynamic association constants of ion-pairs

Table S3 Effective dipole moments (μ) for various ion-pairs (IP), ion-pairs bound to receptor **1** or receptor **1** itself.

Species	Type of IP	μ / D	Method
**1** (open form)		8.22	B3LYP-D3/def2-TZVP
**1** (twisted form)		1.04	B3LYP-D3/def2-TZVP
SrI^+	CIP	32.0	geometric model
	SIP	68.3	geometric model
**1**•SrI^+	CIP	31.6	B3LYP-D3/def2-TZVPD
$SrClO_4^+$	CIP	32.6	geometric model
	SIP	66.8	geometric model
**1**•$SrClO_4^+$	CIP	29.2	B3LYP-D3/def2-TZVPD
BaI^+	CIP	34.7	geometric model
	SIP	67.3	geometric model
**1**•BaI^+	CIP	31.8	B3LYP-D3/def2-TZVPD
$BaClO_4^+$	CIP	35.7	geometric model
	SIP	67.3	geometric model
**1**•$BaClO_4^+$	CIP	32.3	B3LYP-D3/def2-TZVPD

Table S4 Results[a] of fitting the experimental log K_{MX^+} constants of MX^+ ion-pairs, using Eq. 5 (main text).

Species	Type of IP	log $K^0_{MX^+}$	d / nm	C	D
SrI^+	CIP	3.39 ± 0.13	0.333	–9.57 ± 1.92	12.21 ± 2.77
	SIP	1.95 ± 0.03	0.761	–6.27 ± 0.37	5.89 ± 0.47
$SrClO_4^+$	CIP	2.87 ± 0.04	0.353	–4.17 ± 0.57	4.76 ± 0.78
	SIP	1.76 ± 0.03	0.781	–4.81 ± 0.33	4.21± 0.40
$BaClO_4^+$	CIP	2.67 ± 0.04	0.438	–3.90 ± 0.55	4.07± 0.75
	SIP	1.72 ± 0.04	0.866	–4.92 ± 0.42	4.35± 0.53

[a] The fitted parameters are given with their standard errors (±σ).

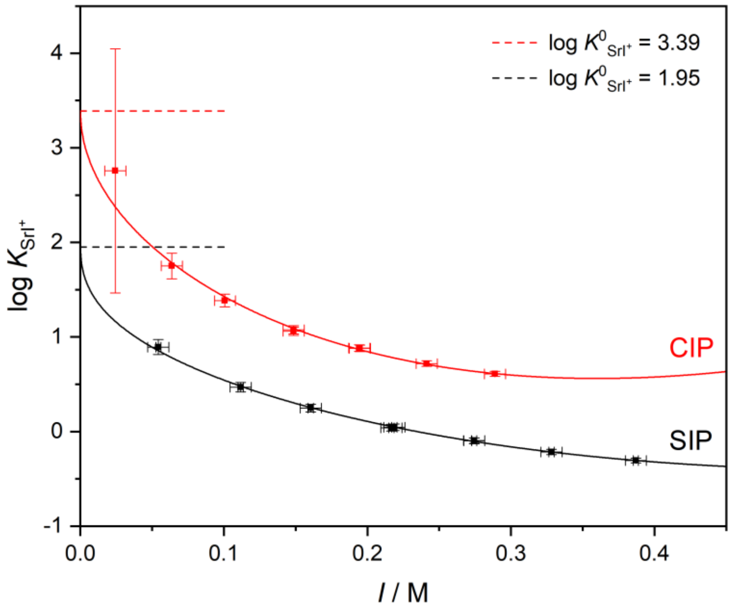

Figure S3 Experimental formation constants (log K_{SrI^+}) assuming the formation of contact (CIP, red symbols) or solvent-separated (SIP, black symbols) SrI^+ ion-pairs as a function of ionic strength (I). Solid lines show fits using Eq. 5 (main text); error bars were calculated assuming error bars were calculated assuming $\sigma(S_{MX^+})$ = ±0.3. Dashed lines indicate the thermodynamic constants (log $K^0_{SrI^+}$) at infinite dilution.

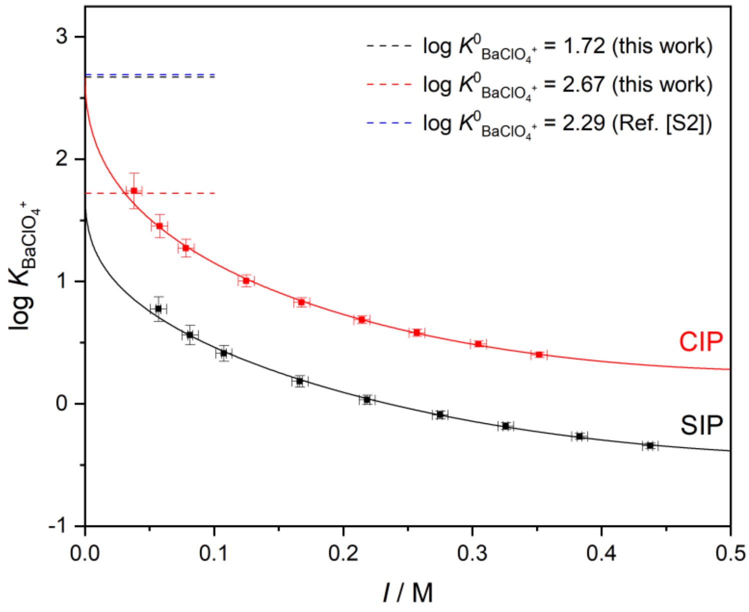

Figure S4 Experimental formation constants (log $K_{BaClO_4^+}$) assuming the formation of contact (CIP, red symbols) or solvent-separated (SIP, black symbols) $BaClO_4^+$ ion-pairs as a function of ionic strength (I). Solid lines show fits using Eq. 5 (main text); error bars were calculated assuming $\sigma(S_{MX^+})$ = ±0.3. Dashed lines indicate the thermodynamic constants (log $K^0_{BaClO_4^+}$) at infinite dilution, obtained in this work or reported in Ref. [S2].

Dielectric relaxation spectrum for a solution of receptor 1

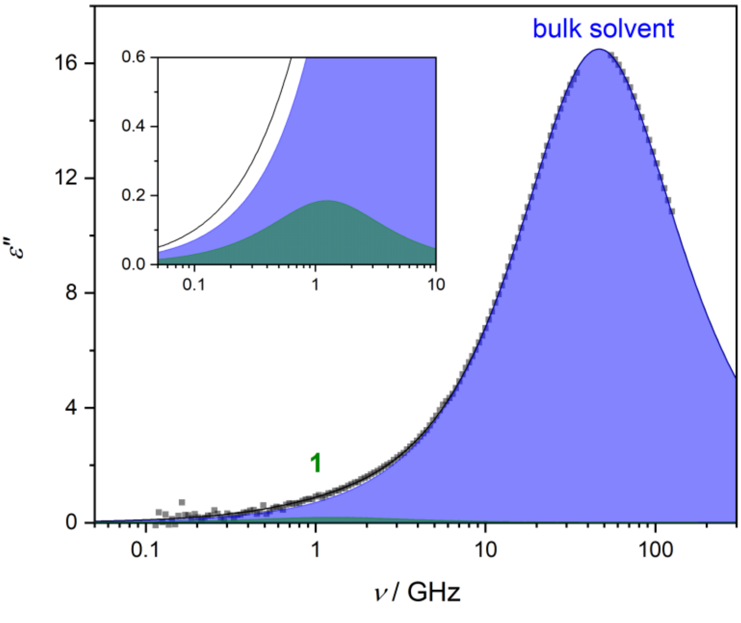

Figure S5 Contribution of acetonitrile (blue-shaded area) and receptor (green-shaded area) to the dielectric loss spectrum (ε'') of a 0.05 M solution of receptor **1**. Squares represent the experimental data, and the black solid line is the result of the fit using Eq. 2 (main text). The inset depicts the zoomed region of the receptor mode for visual clarity.

Parameters of the crystal structures of the [**1**•Sr(ClO_4)$_2$] and [**1**•Ba(ClO_4)$_2$] complexes

The crystal data and refined parameters for the [**1**•Sr(ClO_4)$_2$]·CH_3OH·H_2O and [**1**•Ba(ClO_4)$_2$]$_2$·2H_2O complexes are listed in Tables S5 and S6. The three-dimensional represenation of the structures are shown in Figures 4 and S6.

Table S5 Crystal data (±σ) and structure refinement for [**1**•Sr(ClO_4)$_2$]·CH_3OH·H_2O.

CCDC number	1992910
Empirical formula	$C_{24}H_{31}Cl_7N_6O_{18}Sr$
Formula weight	1027.32
Temperature	173.15 K
Wavelength	0.71073 Å
Crystal system	Triclinic
Space group	P^{-1}
Unit cell dimensions	a = (8.6231 ± 12) Å; α = (87.642 ± 4)°;
	b = (10.4065 ± 15) Å; β = (86.654 ± 5)°;
	c = (22.338 ± 4) Å; γ = (76.079 ± 4)°
Volume	1941.6(5) Å^3
Z	2
Density (calculated)	1.757 Mg/m^3
Absorption coefficient	1.951 mm^{-1}
F(000)	1036
Crystal size	0.231 x 0.214 x 0.067 mm^3
Theta range for data collection	0.913 to 27.483°
Index ranges	$-11 \leq h \leq 11, -13 \leq k \leq 13, -28 \leq l \leq 28$
Reflections collected	26336
Independent reflections	8836 [$R(int)$ = 0.0317]
Completeness to theta = 25.242°	99.3%
Absorption correction	Semi-empirical from equivalents
Max. and min. transmission	1.00000 and 0.84369
Refinement method	Full-matrix least-squares on F^2
Data / restraints / parameters	8836 / 45 / 577
Goodness-of-fit on F^2	1.084
Final R indices [$I > 2\sigma(I)$]	$R1$ = 0.0732, $wR2$ = 0.1995
R indices (all data)	$R1$ = 0.0797, $wR2$ = 0.2078
Extinction coefficient	n/a
Largest diffraction peak and hole	2.467 and –1.422 e·Å^{-3}

Table S6 Crystal data (±σ) and structure refinement for [**1**•Ba(ClO_4)$_2$]$_2$·2H_2O dimer.

CCDC number	1992911
Empirical formula	$C_{44}H_{52}Ba_2Cl_8N_{12}O_{34}$
Formula weight	1851.25
Temperature	173.15 K
Wavelength	0.71073 Å
Crystal system	Triclinic
Space group	P^{-1}
Unit cell dimensions	a = (14.437 ± 2) Å; α = (90.811 ± 2)°;<br>b = (14.439 ± 2) Å; β = (97.185 ± 2)°;<br>c = (16.844 ± 3) Å; γ = (98.475 ± 3)°.
Volume	3443.7(10) Å^3
Z	2
Density (calculated)	1.785 Mg/m^3
Absorption coefficient	1.545 mm^{-1}
F(000)	1840
Crystal size	0.176 x 0.175 x 0.05 mm^3
Theta range for data collection	1.427 to 27.485°
Index ranges	$-18 \leq h \leq 18, -18 \leq k \leq 18, -21 \leq l \leq 21$
Reflections collected	46986
Independent reflections	15672 [$R(int)$ = 0.0285]
Completeness to theta = 25.242°	99.6%
Absorption correction	Semi-empirical from equivalents
Max. and min. transmission	1.0000 and 0.8469
Refinement method	Full-matrix least-squares on F^2
Data / restraints / parameters	15672 / 18 / 911
Goodness-of-fit on F^2	1.058
Final R indices [$I > 2\sigma(I)$]	$R1$ = 0.0502, $wR2$ = 0.1200
R indices (all data)	$R1$ = 0.0531, $wR2$ = 0.1224
Extinction coefficient	n/a
Largest diffraction peak and hole	2.896 and –2.646 e·Å^{-3}

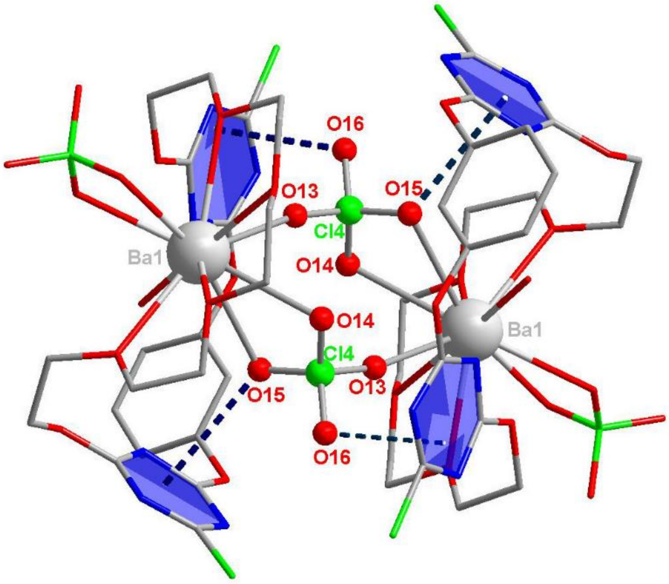

Figure S6 Crystal structure of the [**1**•Ba(ClO_4)$_2$]·2H_2O ion-pair complex. The anion-π distances are indicated by dashed lines connecting the O15/O16 atoms and the blue triazine planes ($d_{O15-plane}$ = 3.063 Å and $d_{O16-plane}$ = 2.944 Å, respectively).

ESI-MS spectra of receptor-containing salt solutions

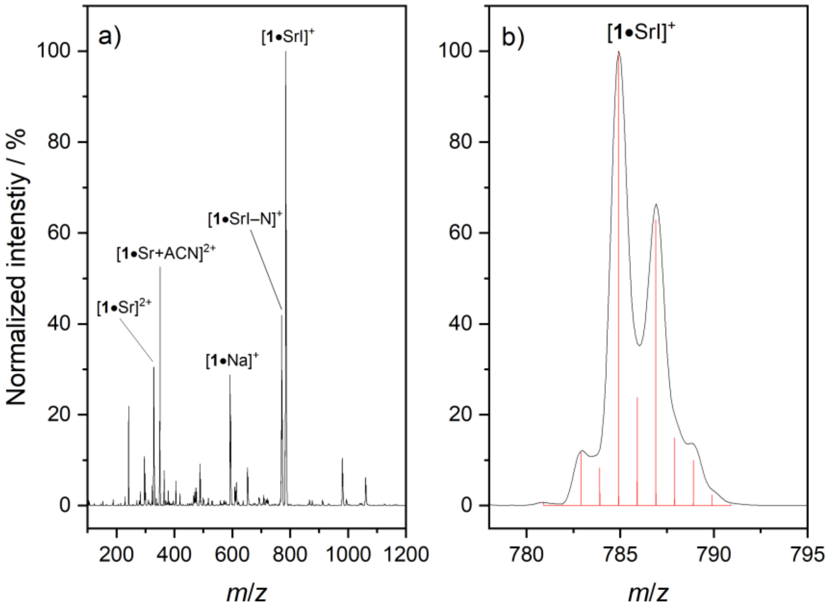

Figure S7 (a) ESI-MS spectrum of a solution containing 0.01 M SrI_2 and 0.01 M receptor **1**, recorded in positive-ion mode. Labels indicate peaks due receptor-bound Sr^{2+}, solvated Sr^{2+}, or SrI^+. –N denotes loss of one N atom. The presence of **1**·Na^+ is due to its trace amounts of sodium in the solvent or to its dissolution from the glass vials. (b) Experimental (black) and calculated (red) spectra for the [**1**•SrI]$^+$ ion ($C_{22}H_{24}Cl_2N_6O_8SrI^+$).

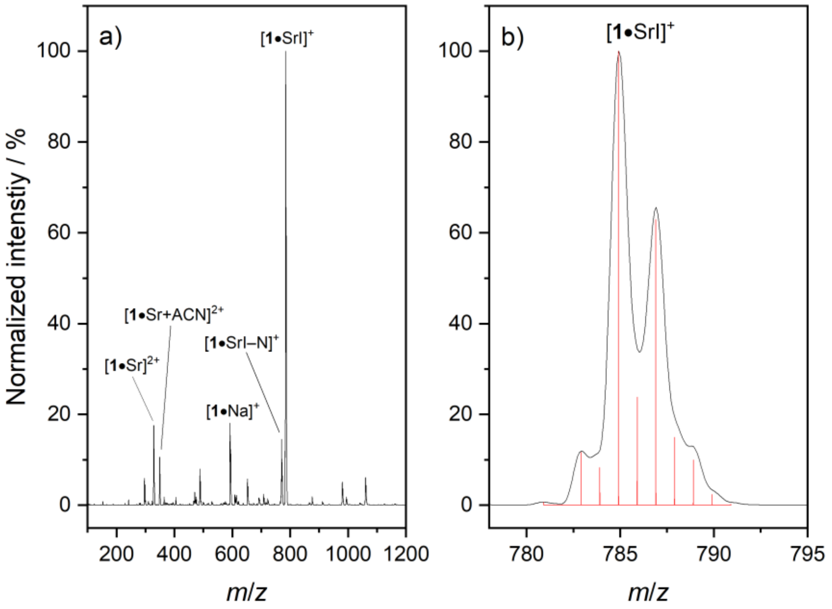

Figure S8 (a) ESI-MS spectrum of a solution containing 0.02 M SrI_2 and 0.02 M receptor **1**, recorded in positive-ion mode. Labels indicate peaks due receptor-bound Sr^{2+}, solvated Sr^{2+}, or SrI^+. –N denotes loss of one N atom. The presence of **1**·Na^+ is due to its trace amounts of sodium in the solvent or to its dissolution from the glass vials. (b) Experimental (black) and calculated (red) spectra for the [**1**•SrI]$^+$ ion ($C_{22}H_{24}Cl_2N_6O_8SrI^+$).

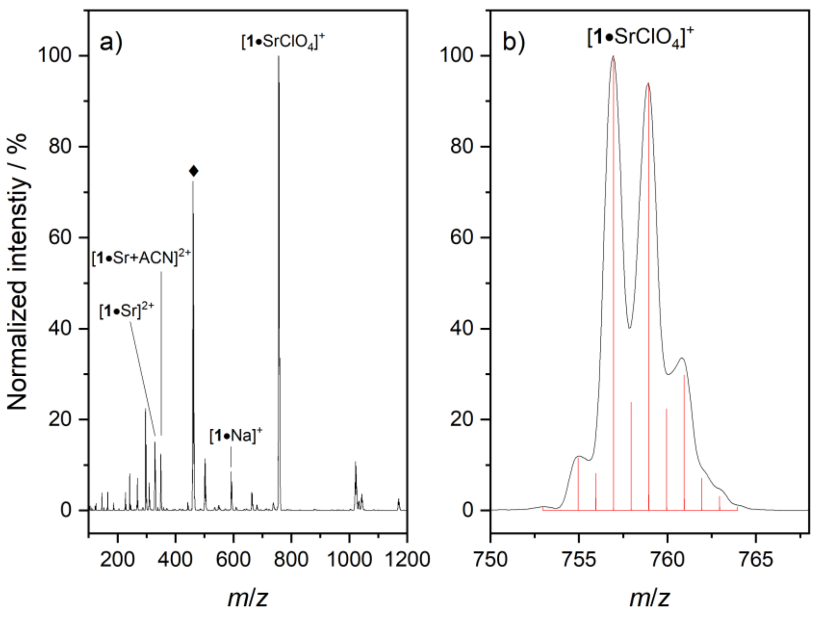

Figure S9 (a) ESI-MS spectrum of a solution containing 0.01 M $Sr(ClO_4)_2$ and 0.01 M receptor **1**, recorded in positive-ion mode. The labels indicate the receptor-bound Sr^{2+}, solvated Sr^{2+}, and $SrClO_4^+$. The presence of **1**·Na^+ is due to its trace amounts of sodium in the solvent or to its dissolution from the glass vials. The diamond shows a fragment forming from the latter species. (b) Experimental (black) and calculated (red) spectra for the [**1**•$SrClO_4$]$^+$ ion ($C_{22}H_{24}Cl_2N_6O_8SrClO_4^+$).

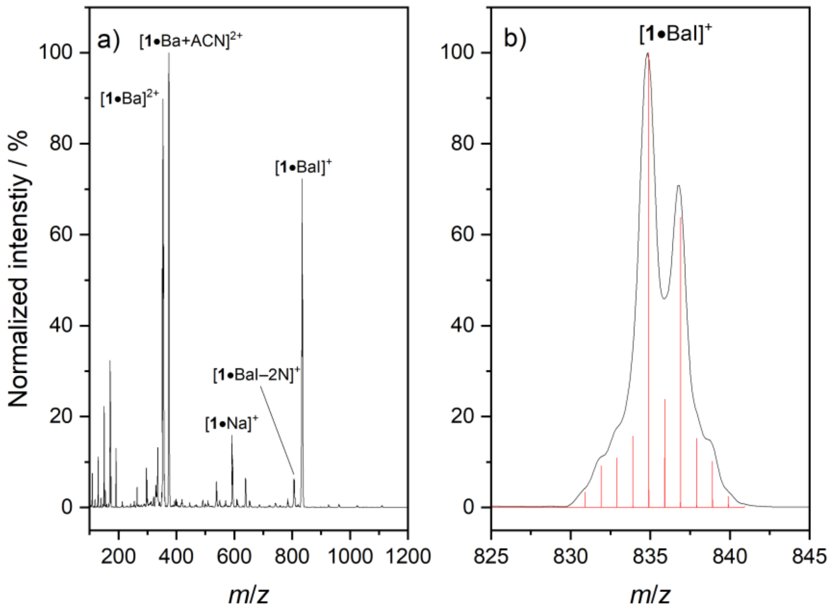

Figure S10 (a) ESI-MS spectrum of a solution containing 0.01 M BaI_2 and 0.01 M receptor **1**, recorded in positive-ion mode. The labels indicate the receptor-bound Ba^{2+}, solvated Ba^{2+}, BaI^+. –2N denotes a loss of two N atoms. The presence of **1**·Na^+ is due to its trace amounts of sodium in the solvent or to its dissolution from the glass vials. (b) Experimental (black) and calculated (red) spectra for the [**1**•BaI]$^+$ ion ($C_{22}H_{24}Cl_2N_6O_8BaI^+$).

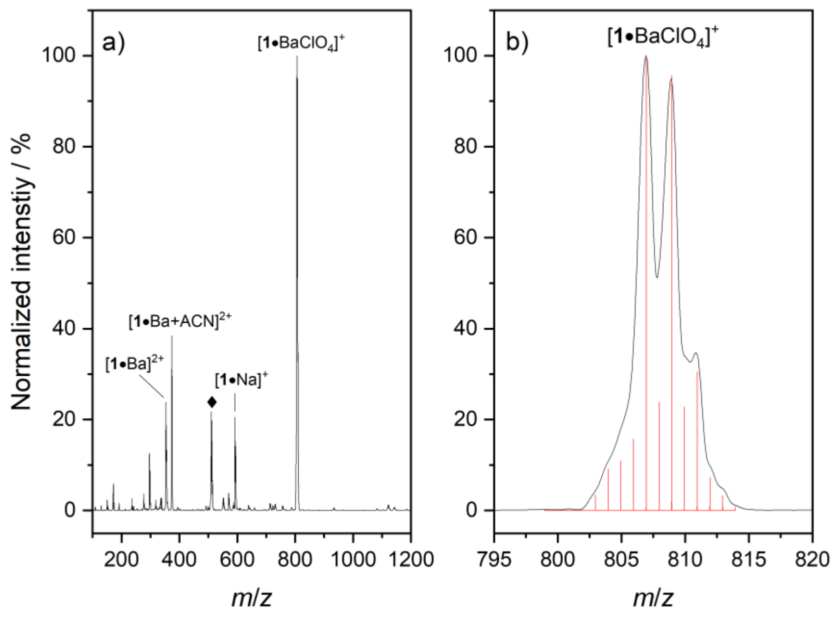

Figure S11 (a) ESI-MS spectrum of a solution containing 0.01 M $Ba(ClO_4)_2$ and 0.01 M receptor **1**, recorded in positive-ion mode. Labels indicate the receptor-bound Ba^{2+}, solvated Ba^{2+}, $BaClO_4^+$. The presence of **1**·Na^+ is due to its trace amounts of sodium in the solvent or to its dissolution from the glass vials. The diamond shows a fragment forming from the latter species. (b) Experimental (black) and calculated (red) spectra for the [**1**•$BaClO_4$]$^+$ ion ($C_{22}H_{24}Cl_2N_6O_8BaClO_4^+$).

DFT optimized structures for the receptor-bound ion-pairs

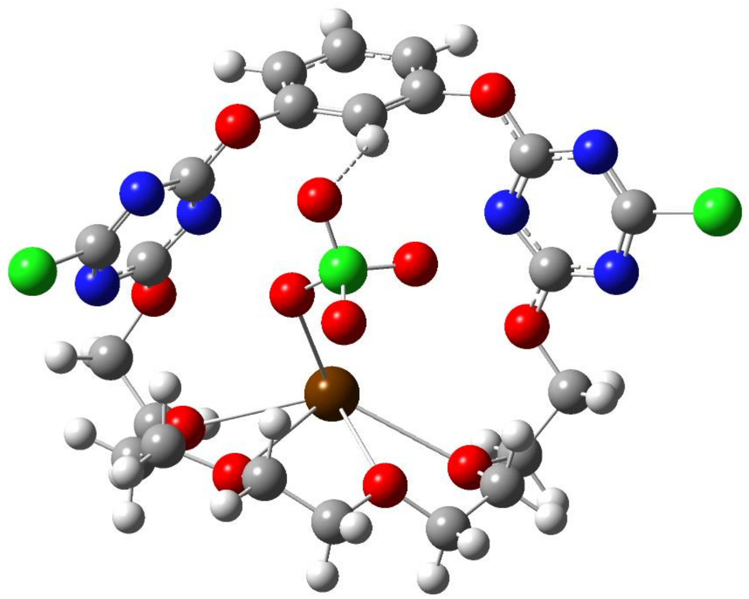

Figure S12 Structure of the $BaClO_4^+$ ion-pair bound to receptor **1**, optimized at the B3LYP-D3/def2-TZVPD level. Implicit solvent effects were taken into account applying the CPCM approach. The calculated effective dipole moment is 32.3 D. The dashed line indicates an intramolecular C–H···O hydrogen bond (d = 2.520 Å).

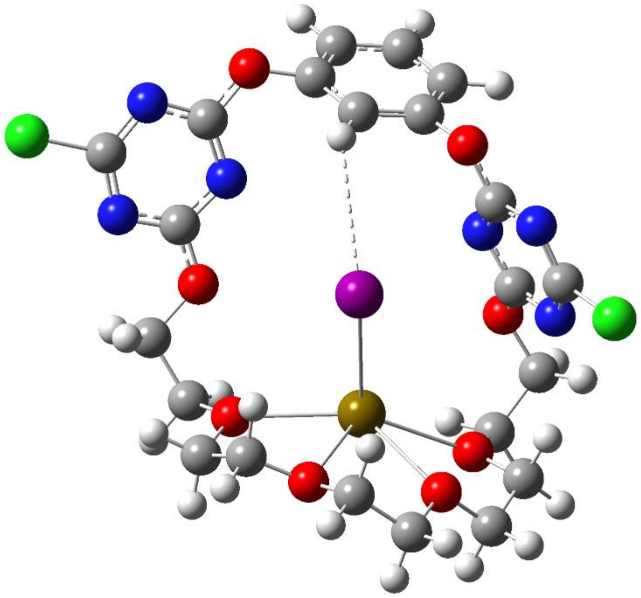

Figure S13 Structure of the SrI^{+} ion-pair bound to receptor **1**, optimized at the B3LYP-D3/def2-TZVPD level. Implicit solvent effects were taken into account applying the CPCM approach. The calculated effective dipole moment is 31.6 D. The dashed line indicates an intramolecular C–H···I^{-} hydrogen bond (d = 3.382 Å).

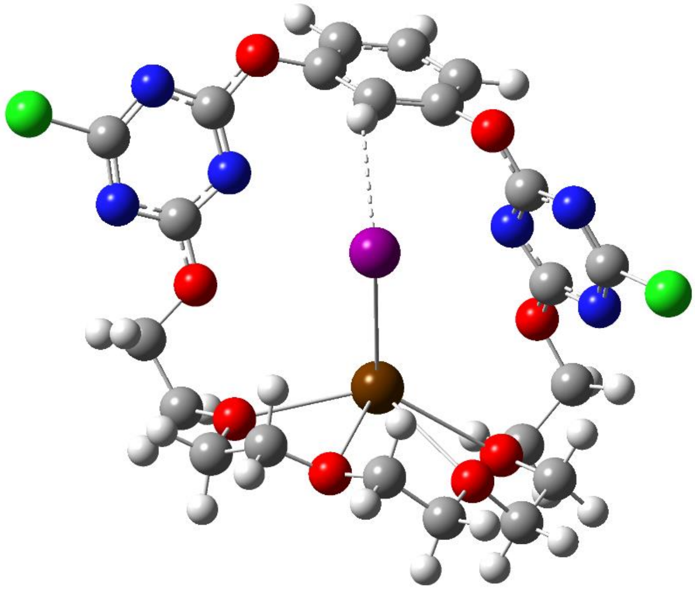

Figure S14 Structure of the BaI^{+} ion-pair bound to receptor **1**, optimized at the B3LYP-D3/def2-TZVPD level. Implicit solvent effects were taken into account applying the CPCM approach. The calculated effective dipole moment is 31.8 D. The dashed line indicates an intramolecular C–H···I^{-} hydrogen bond (d = 3.163 Å).

Dielectric relaxation spectra and relaxation times of receptor-containing salt solutions (varying receptor concentration)

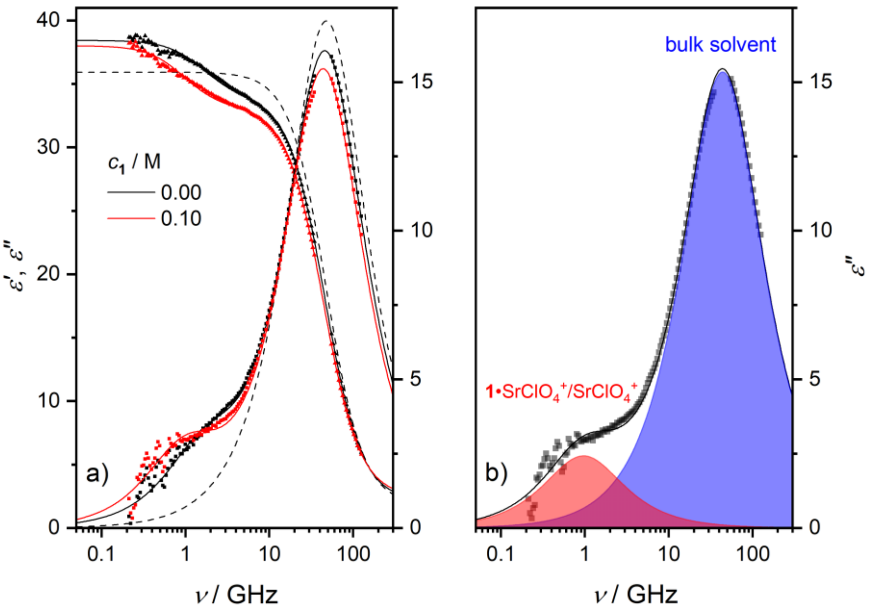

Figure S15 (a) Relative permittivity (*ε'*, triangles, left axis) and dielectric loss (*ε''*, squares, right axis) spectra for 0.10 M $Sr(ClO_4)_2$ solution with (red symbols) and without (black symbols) receptor **1.** Solid lines are the results of fitting Eq. 2 (main text) to the data; dashed line shows the spectrum of acetonitrile, taken from Ref. [S1]. (b) Contribution of acetonitrile (blue-shaded area) and free/receptor-bound $SrClO_4^+$ ion-pairs (red-shaded area) to *ε''* of the solution containing 0.10 M $Sr(ClO_4)_2$ and 0.10 M **1**, as obtained from the fit. Squares represent the experimental data, and the black solid line is the result of the fit. In both panels, the last term of Eq. 2 has been subtracted from *ε''* for visual clarity.

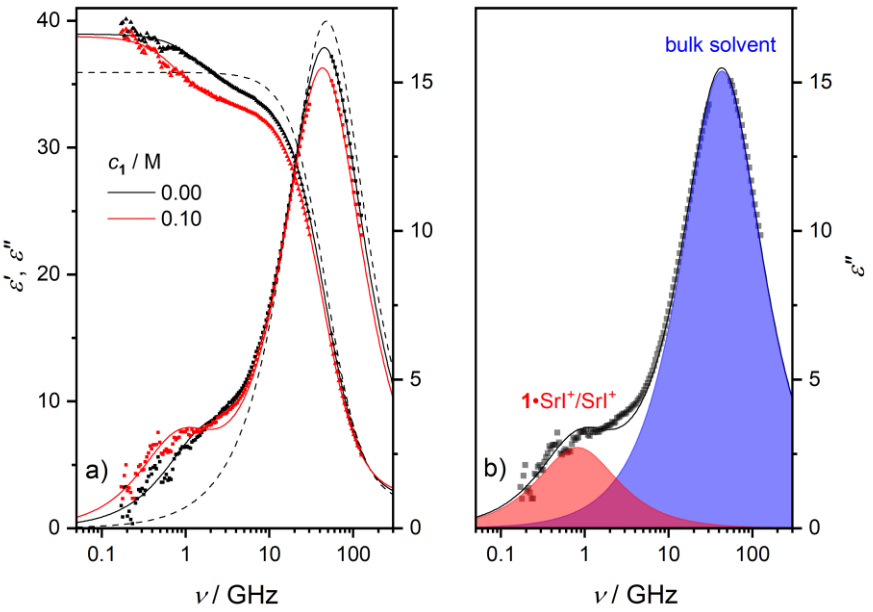

Figure S16 (a) Relative permittivity (*ε'*, triangles, left axis) and dielectric loss (*ε''*, squares, right axis) spectra for a 0.10 M SrI_2 solution with (red symbols) and without (black symbols) receptor **1.** Solid lines are the results of fitting Eq. 2 (main text) to the data; dashed line shows the spectrum of acetonitrile, taken from Ref. [S1]. (b) Contribution of acetonitrile (blue-shaded area) and free/receptor-bound SrI^+ ion-pairs (red-shaded area) to *ε''* of the solution containing 0.10 M SrI_2 and 0.10 M **1**, as obtained from the fit. Squares represent the experimental data, and the black solid line is the result of the fit. In both panels, the last term of Eq. 2 has been subtracted from *ε''* for visual clarity.

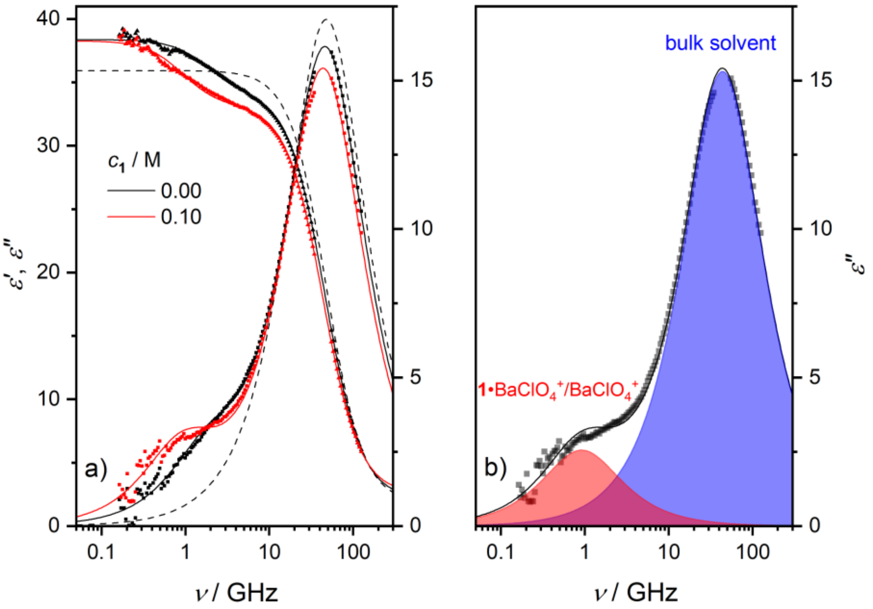

Figure S17 (a) Relative permittivity (ε', triangles, left axis) and dielectric loss (ε'', squares, right axis) spectra for 0.10 M $Ba(ClO_4)_2$ a solution with (red symbols) and without (black symbols) receptor **1.** Solid lines are the results of fitting Eq. 2 (main text) to the data; dashed line shows the spectrum of acetonitrile, taken from Ref. [S1]. (b) Contribution of acetonitrile (blue-shaded area) and free/receptor-bound $BaClO_4^+$ ion-pairs (red-shaded area) to ε'' of the solution containing 0.10 M $Ba(ClO_4)_2$ and 0.10 M **1**, as obtained from the fit. Squares represent the experimental data, and the black solid line is the result of the fit. In both panels, the last term of Eq. 2 has been subtracted from ε'' for visual clarity.

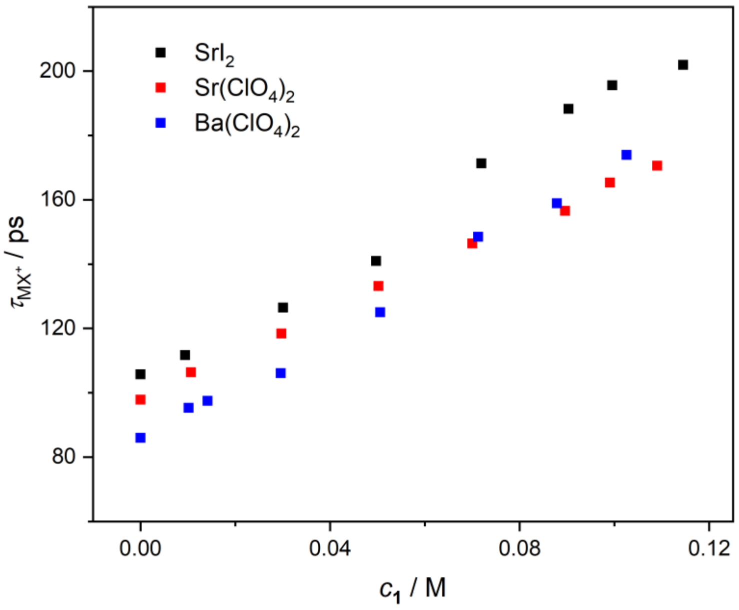

Figure S18 Relaxation time of bare/receptor-bound MX^+ ion-pairs as a function of concentration of receptor **1**, in the presence of 0.10 M SrI_2, $Sr(ClO_4)_2$ or $Ba(ClO_4)_2$.

Determination of the cation- and anion-binding constants via ^{1}H NMR titrations

The ^{1}H NMR spectra were recorded on a Bruker 400 or 500 MHz NMR spectrometer at room temperature ((23 ± 2) °C). All experiments were performed in deuterated acetonitrile; the chemical shifts are reported in ppm and referenced to tetramethylsilane or the residual solvent resonance.

To quantify cation binding, a stock solution containing 0.0105 M **1** was prepared. 500 µL of this solution was placed into an NMR tube sealed with a rubber septum. An initial spectrum was recorded and additional spectra were obtained after aliquots solutions containing $Sr(ClO_4)_2{\cdot}6H_2O$ or $Ba(ClO_4)_2{\cdot}3H_2O$ as well as 0.0105 M **1** were injected sequentially using a microsyringe. To calculate 1:1 cation-binding constants, the chemical shifts of the protons H^a, H^b and H^e (Scheme S1) were used, since these are the most sensitive to cation binding. The data were fitted with the aid of the *PSEQUAD* software [S3].

To study anion binding of **1**, samples containing 0.0105 M **1** and 0.0210 M $Sr(ClO_4)_2{\cdot}6H_2O$ or $Ba(ClO_4)_2{\cdot}3H_2O$ were prepared. Increasing equivalents of Cl^- or I^- anions were added in the form of NBu_4Cl or NBu_4I solutions to the receptor/perchlorate sample and the spectra were recorded after each titration step. To extract the anion binding constants, the peaks of H^a, H^b, H^h and H^f (Scheme S1) were used, with the latter being in particular sensitive to anion coordination [S4]. The binding constants were obtained using the *Bindfit* program [S5].

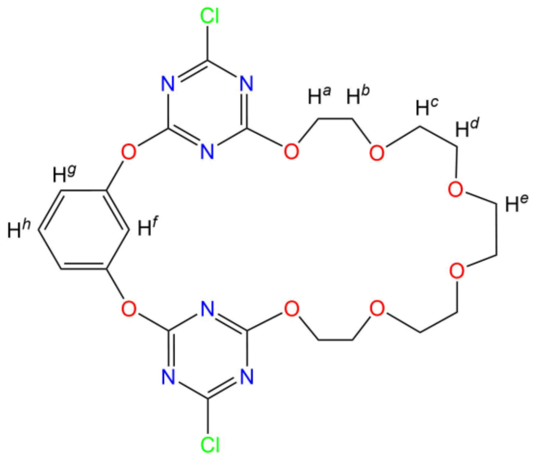

Scheme S1 Structure of ion-pair receptor **1,** indicating all protons having different chemical environment and thus appear at different chemical shifts in the NMR spectrum of **1**.

As will be shown below, the NMR results are mostly sensitive to cation binding. To quantify this equilibrium, we recorded the NMR spectra of 0.01 M **1**, with increasing concentration of $Sr(ClO_4)_2$ (Figure 19a) and $Ba(ClO_4)_2$ (Figure S20a). We observe a gradual downfield shift for the signals of the pentaethylene glycol chain ($H^{a\text{–}e}$), due to the coordination of cations. The variation of peak positions for a given proton (δ) allows for the calculation of the equilibrium constant for the cation binding of **1**, assuming 1:1 binding stoichiometry:

$$K_{\mathbf{1}\bullet\mathrm{M}^{2+}} = \frac{[\mathbf{1}\bullet\mathrm{M}^{2+}]\cdot c^{\emptyset}}{[\mathrm{M}^{2+}]\cdot[\mathbf{1}]} = \frac{[\mathbf{1}\bullet\mathrm{M}^{2+}]\cdot c^{\emptyset}}{(c_{salt}-[\mathbf{1}\bullet\mathrm{M}^{2+}])\cdot(c_{\mathbf{1}}-[\mathbf{1}\bullet\mathrm{M}^{2+}])} \quad \text{(S1)}$$

$$\delta = \delta_{\mathbf{1}}\frac{[\mathbf{1}]}{c_{\mathbf{1}}} + \delta_{\mathbf{1}\bullet\mathrm{M}^{2+}}\frac{[\mathbf{1}\bullet\mathrm{M}^{2+}]}{c_{\mathbf{1}}} \quad \text{(S2)}$$

where species in square brackets denote their equilibrium concentrations, while $\delta_{\mathbf{1}}$ and $\delta_{\mathbf{1}\bullet M^{2+}}$ are their chemical shifts.

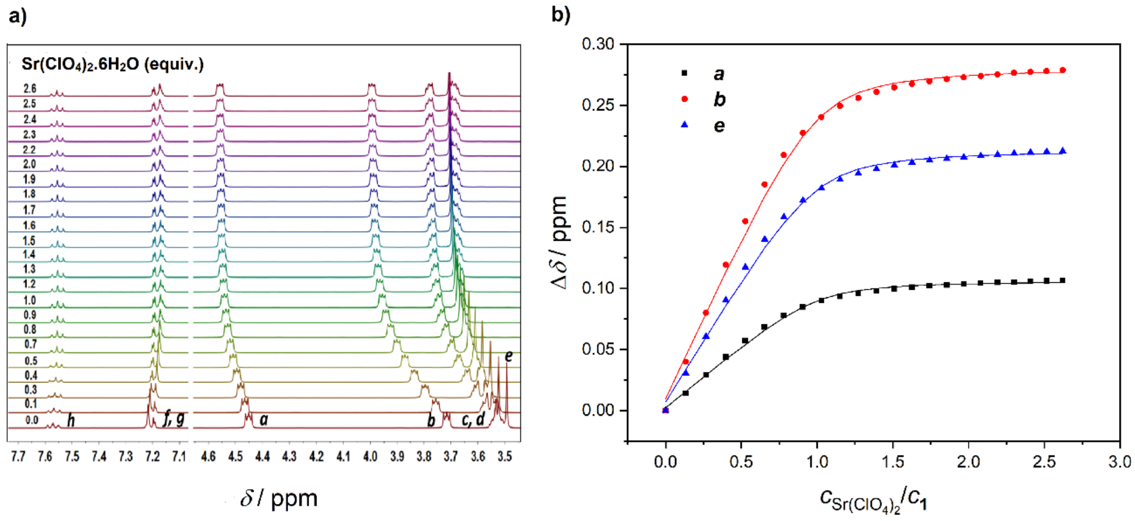

Figure S19 (a) ^{1}H NMR spectra of 0.0105 M receptor **1** in acetonitrile, with increasing equivalents of $Sr(ClO_4)_2$. (b) Increase of the chemical shifts ($\Delta\delta$) of protons H^a, H^b, and H^e of **1** upon addition of $Sr(ClO_4)_2$. Symbols represent experimental data and solid lines show the result of the fit using Eq. S2.

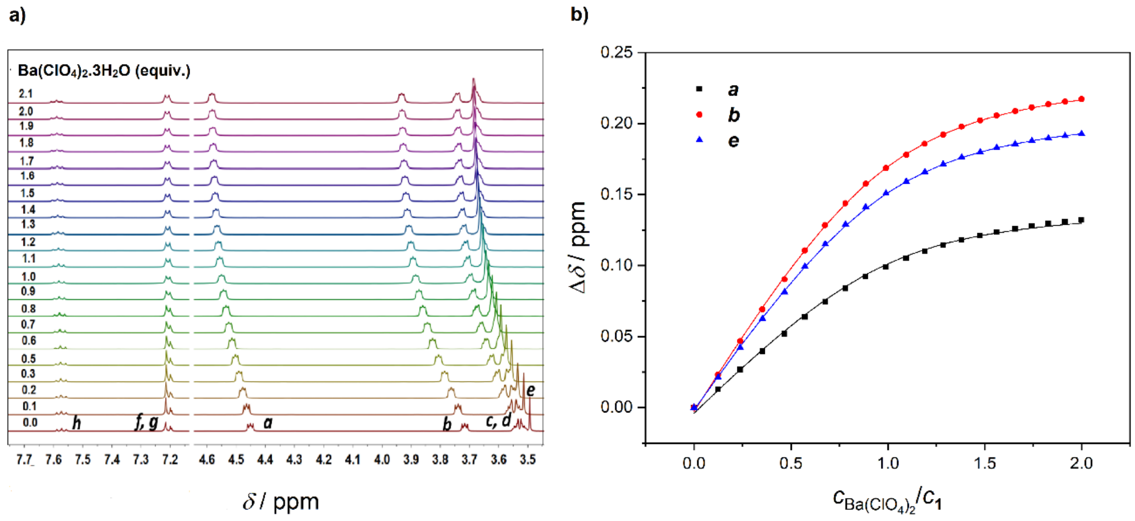

Figure S20 (a) ^{1}H NMR spectra of 0.0105 M receptor **1** in acetonitrile, with increasing equivalents of $Ba(ClO_4)_2$. (b) Increase of the chemical shifts ($\Delta\delta$) of protons H^a, H^b, and H^e of **1** upon addition of $Ba(ClO_4)_2$. Symbols represent experimental data and solid lines show the result of the fit, using Eq. S2.

This model results in an excellent description of the chemical shifts of H^a, H^b and H^e as a function of both $c_{Sr(ClO4)_2}$ (Figure S19b) and $c_{Ba(ClO4)_2}$ (Figure S20b); the calculated log $K_{\mathbf{1}\bullet M^{2+}}$ stability constants are listed in Table S7. Together with data from Ref. [S4], we find log $K_{\mathbf{1}\bullet M^{2+}}$ to follow Sr^{2+} > Ca^{2+} > Ba^{2+} >> Mg^{2+}, which shows that the ionic radius of Sr^{2+} matches the size of the cation binding cavity best.

Since the ESI-MS and also the dielectric data (see the discussion in the main text) provide evidence for the formation of **1**•$MClO_4^+$ complexes, we also tried to account for the formation of **1**•$MClO_4^+$: a model including a subsequent association of a ClO_4^- to **1**•M^{2+} to form **1**•$MClO_4^+$ did not converge, which demonstrates that **1**•M^{2+} and **1**•$MClO_4^+$ species cannot be discriminated based on their ^{1}H NMR chemical shifts. Alternatively, we also tested a model assuming

the exclusive formation of the **1**•$MClO_4^+$ complexes, which however yielded worse fits of the chemical shifts than the **1**•M^{2+} model (see Figures S21 and S22). Consequently, all spectral variations can be formally described by the **1**•M^{2+} complexes, rendering quantification of anion binding from the NMR chemical shifts challenging. This is supported by the observation that the variation of H^f (Figure S19a), to which ClO_4^- forms a weak hydrogen bond (as inferred from the DFT structures), is weak upon addition of $Sr(ClO_4)_2$ or even invariant in the case of $Ba(ClO_4)_2$.

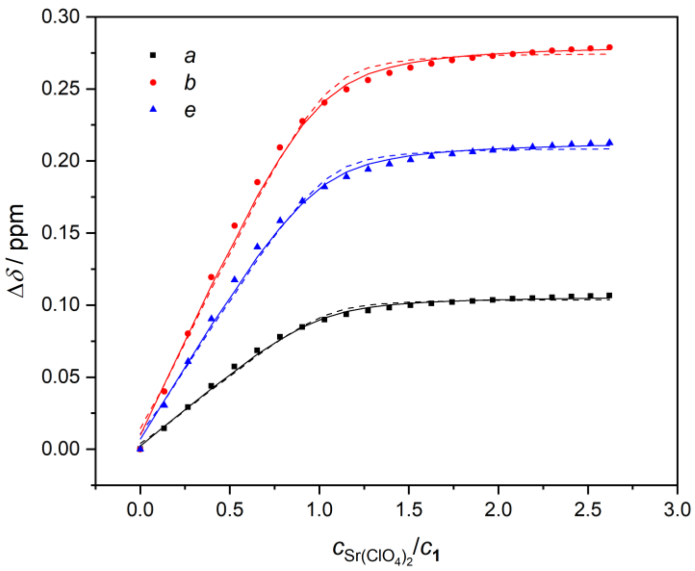

Figure S21 Chemical shift ($\Delta\delta$) of protons H^a, H^b, and H^e of receptor **1** upon addition of $Sr(ClO_4)_2$. Symbols represent experimental data and lines show the result of the fit, assuming the formation of either **1**•Sr^{2+} (solid lines) or **1**•$SrClO_4^+$ (dashed lines). The corresponding fitted equilibrium constants are log $K_{1\bullet Sr^{2+}}$ = 3.48 ± 0.04 and log $K_{1\bullet SrClO_4^+}$ = 5.65 ± 0.07, respectively.

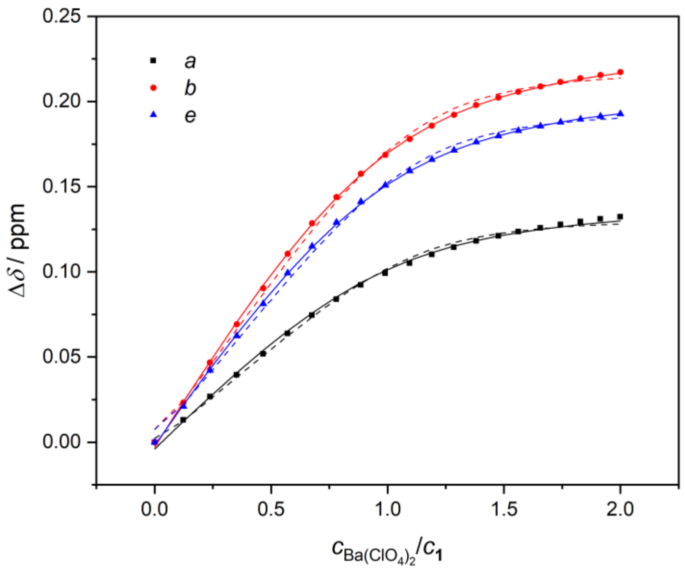

Figure S22 Chemical shift ($\Delta\delta$) of protons H^a, H^b, and H^e of receptor **1** upon addition of $Ba(ClO_4)_2$. Symbols represent experimental data and lines show the result of the fit, assuming the formation of either **1**•Ba^{2+} (solid lines) or **1**•$BaClO_4^+$ (dashed lines). The corresponding fitted equilibrium constants are log $K_{1\bullet Sr^{2+}}$ = 2.90 ± 0.02 and log $K_{1\bullet SrClO_4^+}$ = 5.04 ± 0.03, respectively.

The formation constants of the **1**•M^{2+} complexes are higher than those of the **1**•$MClO_4^+$ species obtained from DRS (Table 2 of the main manuscript). This apparent discrepancy can be rationalized by the notion that contrary to DRS, which is exclusively sensitive to complexes containing one cation and one anion (i.e. **1**•MX^+), the $H^{a–e}$ chemical shifts are predominantly sensitive to cation binding, due to the spatial proximity of the ether chain to the cation. That is, NMR detects both **1**•M^{2+} and **1**•$MClO_4^+$ and the apparent concentrations of [**1**•M^{2+}] in Eq. S1 are the sum of [**1**•M^{2+}] and [**1**•$MClO_4^+$]. As such, only a subset of the NMR detected species containing **1** and M^{2+} are **1**•MX^+ complexes, which explains the higher stability constants obtained from NMR experiments as compared to DRS.

To study iodide binding, we conducted NMR titrations by adding Bu_4NI to samples containing 0.0105 M **1** and 0.0210 M M $Sr(ClO_4)_2$ or $Ba(ClO_4)_2$. Based on the $K_{\mathbf{1}\bullet M^{2+}}$ formation constants, >90% of **1** is already in its complexed form in the starting solutions. The addition of NBu_4I to the solutions of **1**•Sr^{2+} as well as **1**•Ba^{2+} results in a pronounced downfield shift of H^f as a token of iodide binding (see Figures S23a and S24a). Contrary to what we observed in case of the cation binding experiments, we detect higher downfield shift for **1**•Ba^{2+} (~0.3 ppm) as compared to **1**•Sr^{2+} (~0.15 ppm), indicating stronger anion coordination by **1**•Ba^{2+}. This difference is in line with the stronger C–H^f···I^- hydrogen bond for **1**•BaI^+ (3.163 Å) than for **1**•SrI^+ (3.382 Å) as deduced from their DFT structures. Concomitantly, we observe a downfield shift for the H^h aromatic and the $H^{a–e}$ aliphatic protons, the latter implying further anion-enhanced cation binding. Thus, receptor **1** displays positive cooperative binding behavior.

These spectral changes allow for calculating the stability constants assuming stepwise binding of two I^- ions, i.e., the formation of **1**•MI^+ and **1**•MI_2 complexes:

$$K_{I^-} = \frac{[\mathbf{1}\bullet MI^+]\cdot(c^\emptyset)}{[\mathbf{1}\bullet M^{2+}]\cdot[I^-]} \tag{S3}$$

$$K_{2I^-} = \frac{[\mathbf{1}\bullet MI_2]\cdot(c^\emptyset)}{[\mathbf{1}\bullet MI^+]]\cdot[I^-]} \tag{S4}$$

$$\delta = \delta_{\mathbf{1}\bullet M^{2+}}\frac{[\mathbf{1}]}{c_1} + \delta_{\mathbf{1}\bullet MI^+}\frac{[\mathbf{1}\bullet MI^+]}{c_1} + +\delta_{\mathbf{1}\bullet MI_2}\frac{[\mathbf{1}\bullet MI_2]}{c_1} \tag{S5}$$

Here we also assume for the free receptor concentration that [**1**] ≈ 0, that is, $c_1 \approx$ **1**•M^{2+}. We perform a global fit using the chemical shifts of the H^a, H^b, H^h in addition to the most sensitive H^f proton. The thus obtained constants are shown in Table S7, while the results of the fits are depicted in Figures S24b and S24b. These fits show that the above model provides an excellent fit to the experimental data. As such, the binding of I^- takes place stepwise. Furthermore, both binding constants for **1**•Ba^{2+} are consistently higher than those of **1**•Sr^{2+}, thus, iodide is bound stronger to the Ba(II) complex.

Table S7 Stability constants (log $K \pm \sigma$, at 23 ± 2 °C) corresponding to the reactions in the first column, obtained in this work via ^{1}H NMR titrations.

**Reaction**	**log *K***
**1** + Sr^{2+} ⇌ **1**•Sr^{2+}	3.46 ± 0.04
**1**•Sr^{2+} + I^- ⇌ **1**•SrI^+	1.07 ± 0.01
**1**•SrI^+ + I^- ⇌ **1**•SrI_2	0.10 ± 0.03
**1** + Ba^{2+} ⇌ **1**•Ba^{2+}	2.87 ± 0.01
**1**•Ba^{2+} + I^- ⇌ **1**•BaI^+	1.64 ± 0.01
**1**•BaI^{2+} + I^- ⇌ **1**•BaI_2	0.63 ± 0.01

We note that the DRS equilibrium constants for **1**•$SrClO_4^+$ and **1**•$BaClO_4^+$ (Table 2 in the main text) are lower than those of **1**•Sr^{2+} and **1**•Ba^{2+}, obtained from ^{1}H NMR titrations (Table S7). Given that predominantly the chemical shifts of the polyether chain vary upon addition of salt, this difference can be rationalized by the notion that NMR is similarly sensitive to the formation of **1**•M^{2+}, **1**•MX^+ (and possibly that of **1**•MX_2 at high anion concentrations) and thus primarily to cation coordination. Consequently, NMR detects all these all cation-containing complexes and thus yield higher equilibrium concentrations and binding constants than DRS, which detects only dipolar ion-pairs (**1**•MX^+).

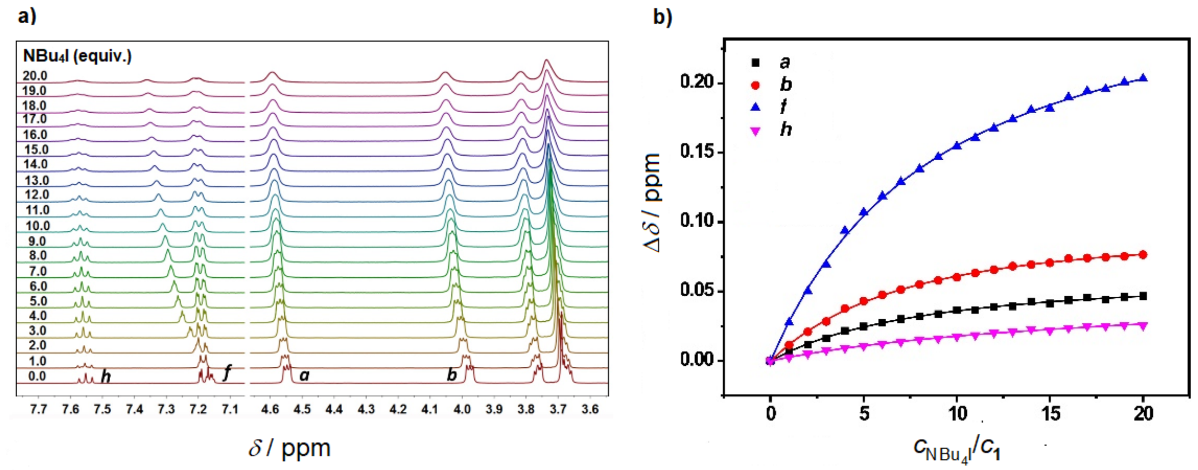

Figure S23 (a) ^{1}H NMR spectra of $1.05{\cdot}10^{-2}$ M receptor **1** + $2.10{\cdot}10^{-2}$ M $Sr(ClO_4)_2{\cdot}6H_2O$ in acetonitrile, with increasing equivalents of Bu_4NI. (b) Chemical shifts ($\Delta\delta$) of protons H^a, H^b, H^e and H^f upon addition of Bu_4NI. Symbols represent experimental data and solid lines show the result of the fit, using Eq. S4.

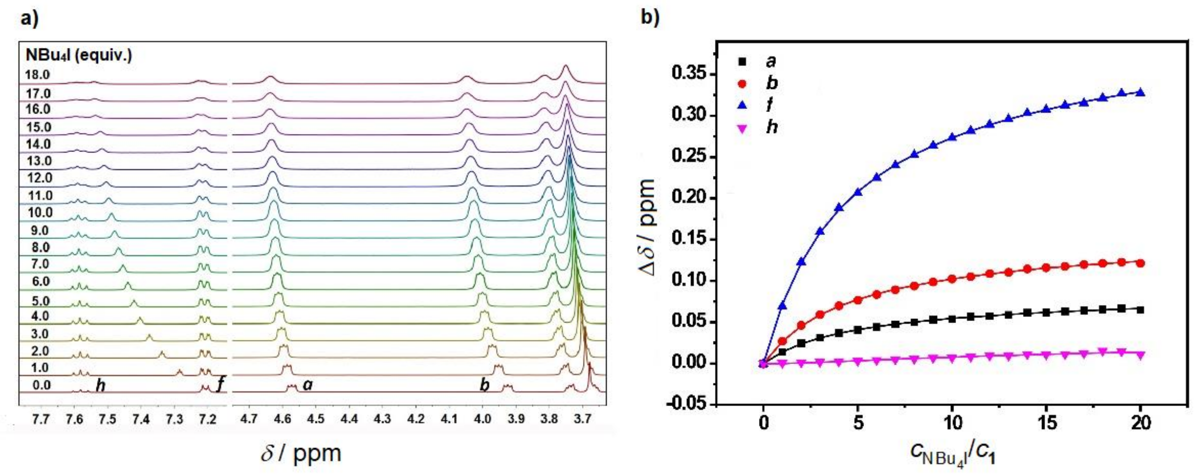

Figure S24 (a) ^{1}H NMR spectra of $1.05{\cdot}10^{-2}$ M receptor **1** + $2.10{\cdot}10^{-2}$ M $Ba(ClO_4)_2{\cdot}3H_2O$ in acetonitrile, with increasing equivalents of Bu_4NI. (b) Chemical shifts ($\Delta\delta$) of protons H^a, H^b, H^e and H^f upon addition of Bu_4NI. Symbols represent experimental data and solid lines show the result of the fit, using Eq. S4.

Determination of the dipole moment of the 1•BaI$^+$ complex

In the main text, we argue that the dipole moments of the bare ion-pairs and the receptor-bound ion-pairs are similar. To support this notion experimentally, we perform experiments on solutions containing 0.01–0.05 M BaI_2 and 0.05 M **1**. As BaI_2 is hardly soluble in acetonitrile but can be solubilized in the presence of **1**, the formation of bare BaI$^+$ IPs can be ruled out and therefore the solute relaxation mode detected in the DRS spectra (Figure S25) is solely due to the formation of receptor-bound ion-pairs. The finite conductivity of the samples provides evidence for dissociated ions, suggesting the electroneutral **1**•BaI_2 to form only to a minor extent (Figure S26). Assuming thus the exclusive formation of **1**•BaI$^+$, the effective dipole moment of the **1**•BaI$^+$ complexes ($\mu_{\mathbf{1}\cdot\mathrm{BaI}^+}$) can be directly obtained from the corresponding relaxation amplitude ($S_{\mathbf{1}\cdot\mathrm{BaI}^+}$) via Eq. 3 (see the main text). For these calculations, we assume the cavity-field factor of **1**•BaI$^+$ to be the same as that of BaI$^+$, the latter is obtained from the geometric model described in Ref. [S6]. To estimate the standard error (σ) of $\mu_{\mathbf{1}\cdot\mathrm{BaI}^+}$, we assume $\sigma(S_{\mathbf{1}\cdot\mathrm{BaI}^+})$ to be ±0.3. The thus obtained dipole moments are shown in Figure S27.

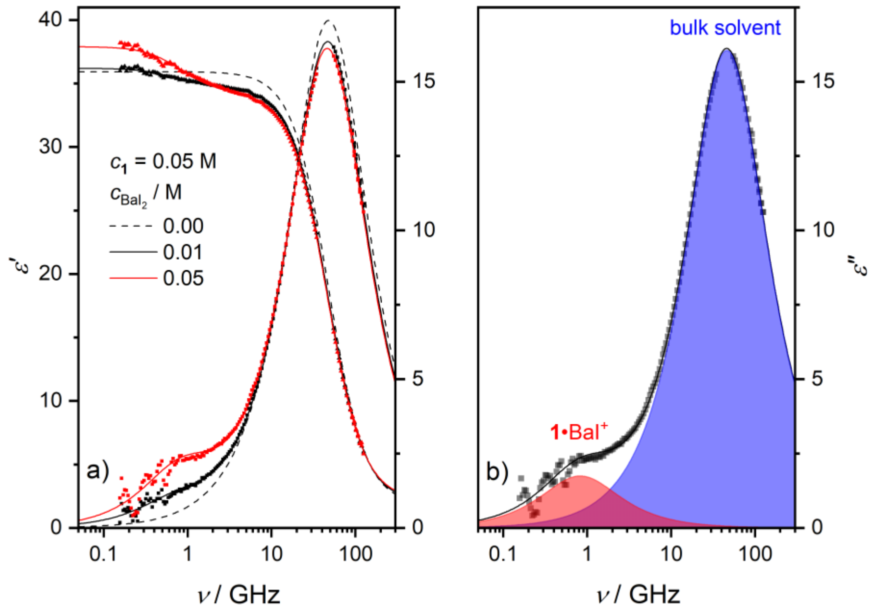

Figure S25 (a) Relative permittivity (ε', triangles, left axis) and dielectric loss (ε'', squares, right axis) spectra of 0.01–0.05 M BaI_2 solutions containing 0.05 M receptor **1**. Solid lines are the results of fitting Eq. 2 to the data; dashed line shows the spectrum of pure acetonitrile, taken from Ref. [S1]. (b) Contribution of acetonitrile (blue-shaded area) and **1**•BaI$^+$ ion-pairs (red-shaded area) to ε'' of the solution containing 0.05 M BaI_2 and 0.05 M **1**, as obtained from the fit. Squares represent the experimental data, and the black solid line is the result of the fit. In both panels, the last term of Eq. 2 has been subtracted from ε'' for visual clarity.

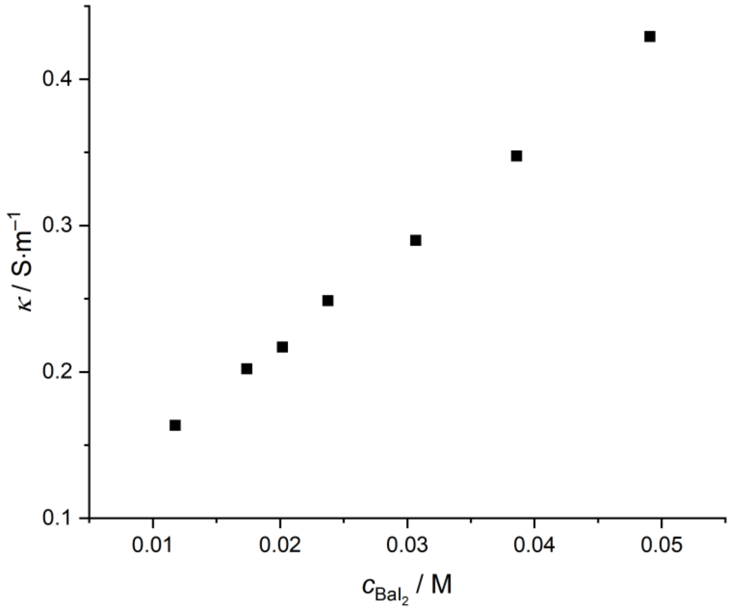

Figure S26 Conductivities of 0.01–0.05 M BaI_2 solutions in the presence of 0.05 M receptor **1**. The conductivities were obtained from fitting Eq. 2 to the dielectric spectra.

This analysis yields an average value of $\mu_{\mathbf{1}\bullet\mathrm{BaI}^+}$ = (34 ± 4) D, which agrees well with the value obtained from the DFT structure optimization (31.8 D, Table S3) and also with that of the bare BaI^+ species (34.7 D, Table S3). Thus, this comparison shows that the electrical dipole moment of the bare ion-pairs and the receptor pound ion-pairs are very similar, indicative of similar cation-anion separation.

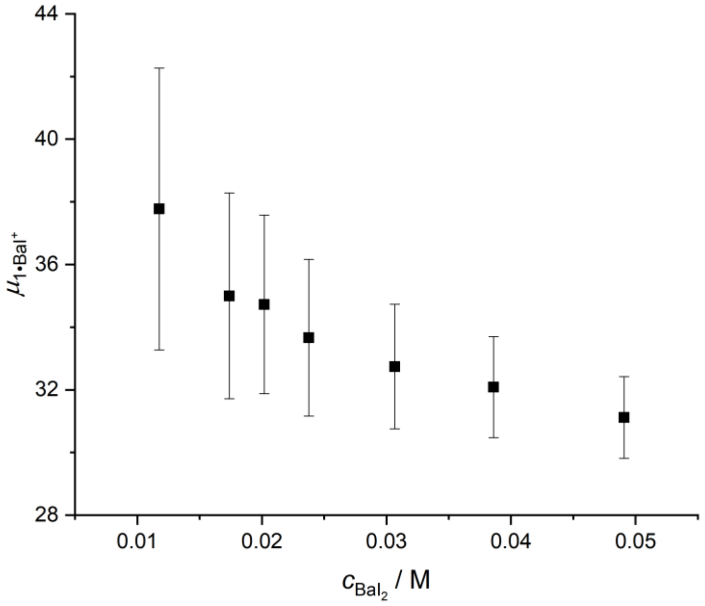

Figure S27 Calculated dipole moments for the **1**•BaI^+ species for solutions containing 0.01–0.05 M BaI_2. The dipole moments and their error bars were obtained via Eq. 3 (main text), assuming $\sigma(S_{\mathbf{1}\bullet\mathrm{BaI}^+}) = \pm 0.3$.

Conductivities of receptor-containing salt solutions (varying receptor concentration)

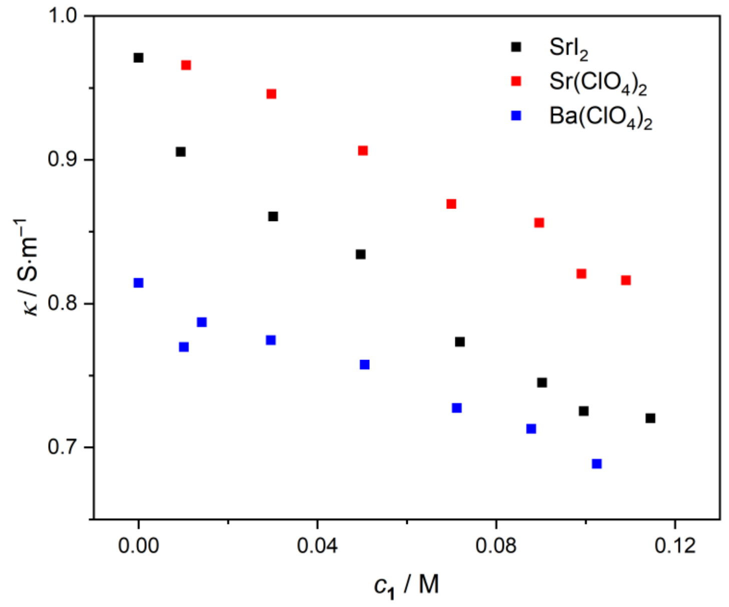

Figure S28 Conductivities of 0.10 M SrI_2, $Sr(ClO_4)_2$ and $Ba(ClO_4)_2$ solutions as a function of concentration of receptor **1**. The conductivities were obtained from fitting Eq. 2 (main text) to the dielectric spectra.

Relaxation times, conductivities and ion-pair concentrations for receptor-containing salt solutions (varying salt concentration)

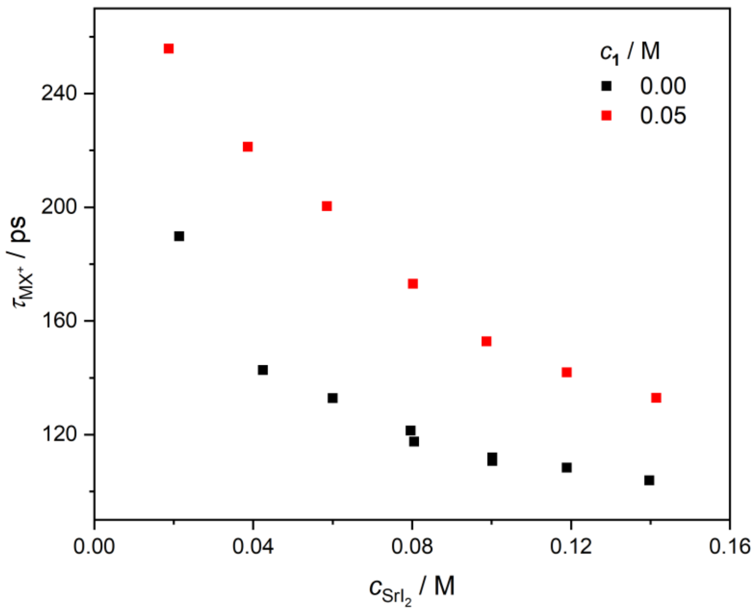

Figure S29 Relaxation times of bare/receptor-bound MX^+ ion-pairs for solutions of 0.02–0.14 M SrI_2 with and without 0.05 M receptor **1**. The relaxation times were obtained from fitting Eq. 2 (main text) to the dielectric spectra. The experiments from these series demonstrates the longer relaxation times in the presence of **1**.

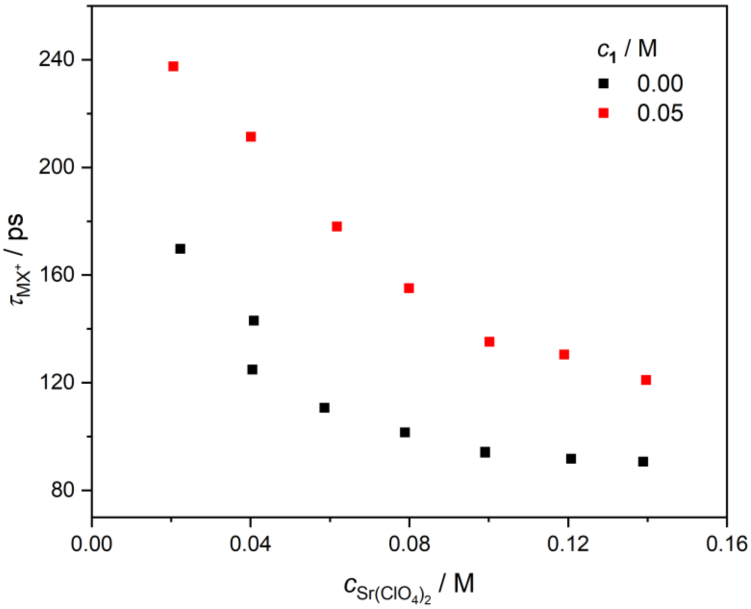

Figure S30 Relaxation times of bare/receptor-bound MX^+ ion-pairs for solutions of 0.02–0.14 M $Sr(ClO_4)_2$ with and without 0.05 M receptor **1**. The relaxation times were obtained from fitting Eq. 2 (main text) to the dielectric spectra. The experiments from these series demonstrates the longer relaxation times in the presence of **1**.

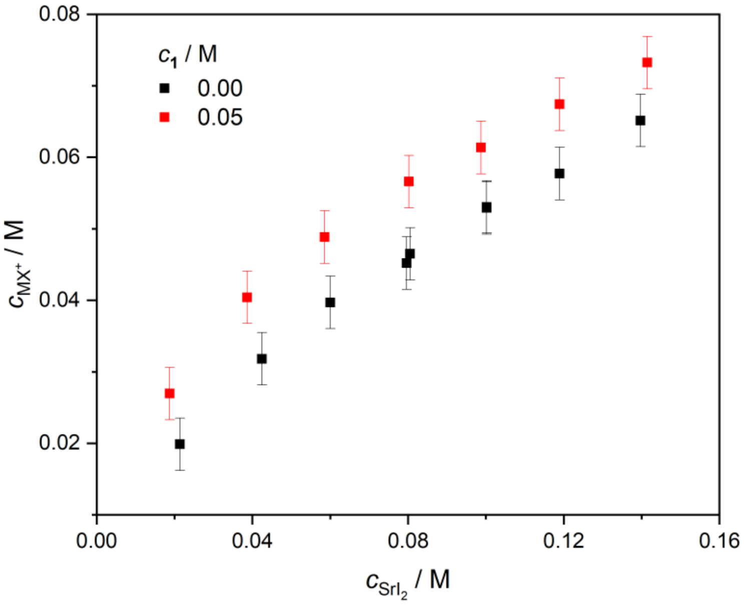

Figure S31 Concentrations of the bare/receptor-bound MX^+ ion-pairs for 0.02–0.14 M SrI_2 solutions with and without 0.05 M receptor **1**. The concentrations and their error bars were calculated via Eq. 3 (main text), assuming $\sigma(S_{MX^+}) = \pm 0.3$.

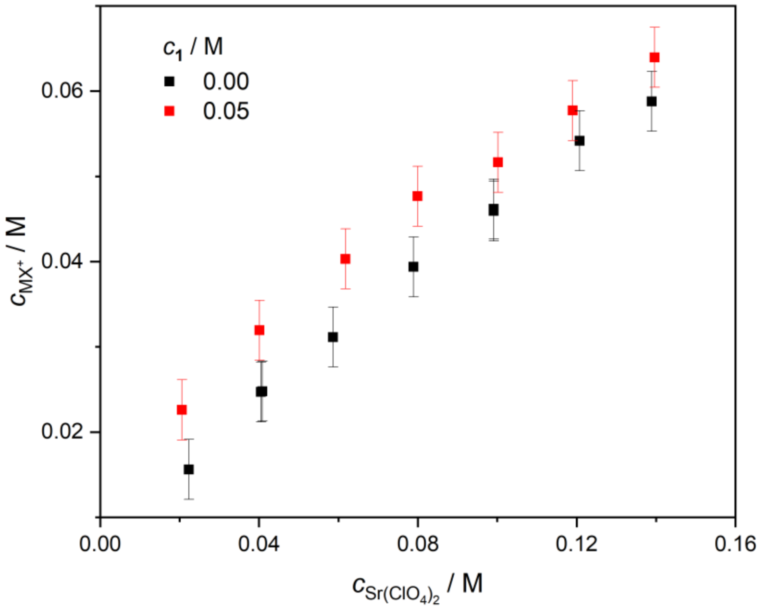

Figure S32 Concentrations of the bare/receptor-bound MX^+ ion-pairs for 0.02–0.14 M $Sr(ClO_4)_2$ solutions with and without 0.05 M receptor **1**. The concentrations and their error bars were calculated via Eq. 3 (main text), assuming $\sigma(S_{MX^+}) = \pm 0.3$.

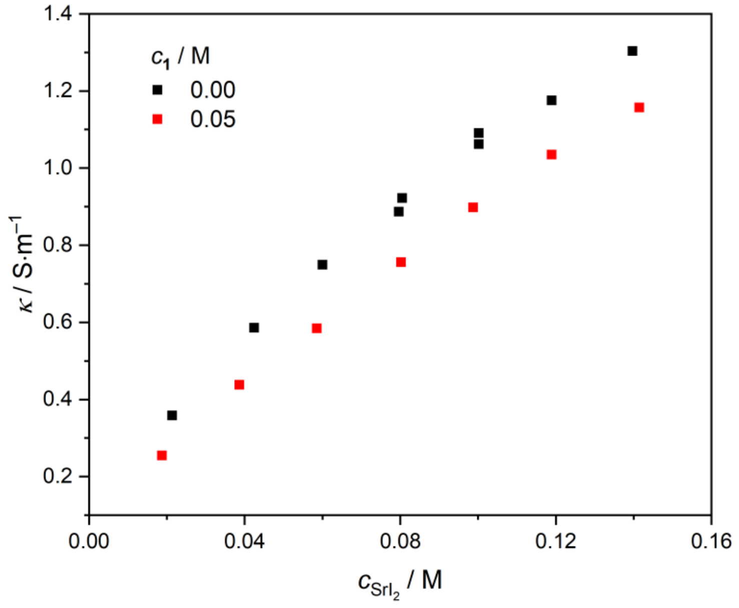

Figure S33 Conductivities of the of 0.02–0.14 M SrI_2 solutions with and without 0.05 M receptor **1**. The conductivities were obtained from fitting Eq. 2 (main text) to the dielectric spectra. The reduced conductivity in the presence of **1** evidence enhanced ion-pairing in the presence of the receptor.

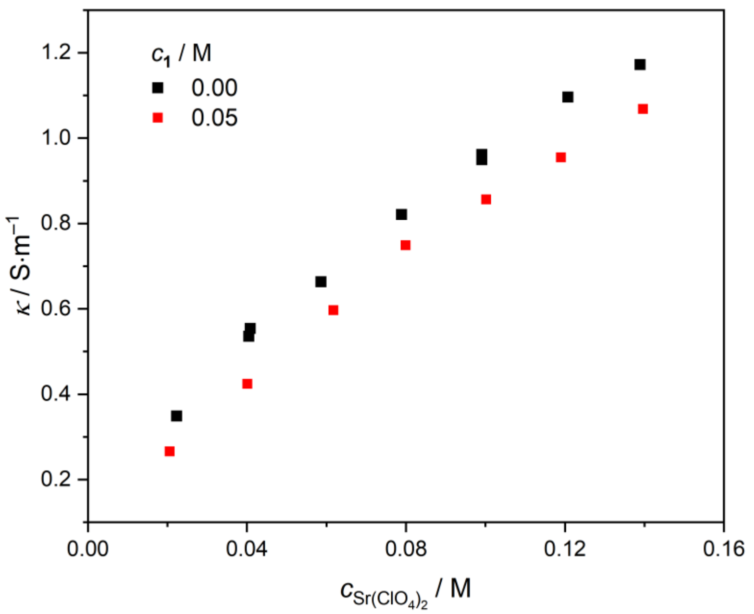

Figure S34 Conductivities of the of 0.02–0.14 M $Sr(ClO_4)_2$ solutions with and without 0.05 M receptor **1**. The conductivities were obtained from fitting Eq. 2 (main text) to the dielectric spectra. The reduced conductivity in the presence of **1** evidence enhanced ion-pairing in the presence of the receptor.

References

[S1] P. Eberspächer, E. Wismeth, R. Buchner, J. Barthel, *J. Mol. Liq*. **2006**, *129*, 3–12.

[S2] O. N. Kalugin, V. N. Agieienko, N. A. Otrosho, *J. Mol. Liq*. **2012**, *165*, 78–86.

[S3] L. Zékány, I. Nagypál, G. Peintler, PSEQUAD for Chemical Equilibria, Update 5–5.10., Szeged, Debrecen (Hungary), **2008**. Originally published in *Computational Methods for the Determination of Formation Constants* (Ed.: D. J. Leggett), Plenum Press, New York, **1985**, pp. 291–353.

[S4] J. Luo, Y.-F. Ao, C. Malm, J. Hunger, Q.-Q. Wang, D.-X. Wang, *Dalton Trans*. **2018**, *47*, 7883–7887

[S5] Bindfit, V. 05. The program is based on the following publication: P. Thordarson, *Chem. Soc. Rev*. **2011**, *40*, 1305–1323.

[S6] J. Barthel, H. Hetzenauer, R. Buchner, *Ber. Bunsenges. Phys. Chem*. **1992**, *96*, 1424–1432.
